# All-solution-processed ultraflexible wearable sensor enabled with universal trilayer structure for organic optoelectronic devices

**DOI:** 10.1126/sciadv.adk9460

**Published:** 2024-04-10

**Authors:** Lulu Sun, Jiachen Wang, Hiroyuki Matsui, Shinyoung Lee, Wenqing Wang, Shuyang Guo, Hongting Chen, Kun Fang, Yoshihiro Ito, Daishi Inoue, Daisuke Hashizume, Kazuma Mori, Masahito Takakuwa, Sunghoon Lee, Yinhua Zhou, Tomoyuki Yokota, Kenjiro Fukuda, Takao Someya

**Affiliations:** ^1^ Thin-Film Device Laboratory, RIKEN, 2-1 Hirosawa, Wako, Saitama 351-0198, Japan.; ^2^ RIKEN Center for Emergent Matter Science (CEMS), 2-1 Hirosawa, Wako, Saitama 351-0198, Japan.; ^3^ Department of Electrical Engineering and Information Systems, The University of Tokyo, 7-3-1 Hongo, Bunkyo-ku, Tokyo 113-8656, Japan.; ^4^ Research Center for Organic Electronics (ROEL), Yamagata University, 4-3-16 Jonan, Yonezawa, Yamagata 992-8510, Japan.; ^5^ Nano Medical Engineering Laboratory, RIKEN Cluster for Pioneering Research, 2-1 Hirosawa, Wako, Saitama 351-0198, Japan.; ^6^ Institute of Engineering Innovation, Graduate School of Engineering, The University of Tokyo, 7-3-1 Hongo, Bunkyo-ku, Tokyo 113-8656, Japan.; ^7^ Wuhan National Laboratory for Optoelectronics, Huazhong University of Science and Technology, Wuhan 430074, China.

## Abstract

All-solution-processed organic optoelectronic devices can enable the large-scale manufacture of ultrathin wearable electronics with integrated diverse functions. However, the complex multilayer-stacking device structure of organic optoelectronics poses challenges for scalable production. Here, we establish all-solution processes to fabricate a wearable, self-powered photoplethysmogram (PPG) sensor. We achieve comparable performance and improved stability compared to complex reference devices with evaporated electrodes by using a trilayer device structure applicable to organic photovoltaics, photodetectors, and light-emitting diodes. The PPG sensor array based on all-solution-processed organic light-emitting diodes and photodetectors can be fabricated on a large-area ultrathin substrate to achieve long storage stability. We integrate it with a large-area, all-solution-processed organic solar module to realize a self-powered health monitoring system. We fabricate high-throughput wearable electronic devices with complex functions on large-area ultrathin substrates based on organic optoelectronics. Our findings can advance the high-throughput manufacture of ultrathin electronic devices integrating complex functions.

## Teaser

A self-powered wearable sensor was developed by integrating all-solution–processed trilayer structure organic optoelectronics.

## INTRODUCTION

The large-scale production of wearable electronic devices is highly desirable owing to the need for commercialization (*1*). In general, wearable electronics attached to a human body or textiles require a combination of different functions (*2*–*4*). The tunable chemical structures and energy levels of organic semiconductors enable them to satisfy the diverse functional requirements of wearable devices. These requirements include photodetection, energy harvesting, and electroluminescence (*5*–*10*). Ultraflexible organic optoelectronic devices based on ultrathin substrates have been successfully developed to achieve a skin-like display and self-powered health monitoring wearable electronics by integrating ultraflexible organic photovoltaics (OPVs) (*11*–*13*), organic light-emitting diodes (OLEDs) (*14*, *15*), and organic photodetectors (OPDs) (*16*, *17*) owing to their excellent mechanical flexibility.

The large-scale production of such flexible organic optoelectronic devices can be achieved via all-solution processing without requiring vacuum processes such as vacuum magnetron sputtering and thermal evaporation techniques (*18*, *19*). All-solution processing enables each layer in organic optoelectronics, from the bottom to the top electrode, to be solution-processed, rendering it compatible with large-scale production, such as roll-to-roll printing (*20*). The recently reported fully handwriting perovskite optoelectronic devices use all-solution processing, making the devices adaptable to diverse substrates with freely fabricated device shapes and ease of operation (*21*). All-solution processing exhibits great potential for scaling up organic and perovskite semiconductor electronic devices (*22*–*25*).

Organic optoelectronic devices typically exhibit similar vertical multilayer-stacking structures, including an anode, a cathode, an electron transport layer (ETL), a hole transport layer (HTL), and organic semiconductors (*26*–*28*). Consequently, wearable sensors can be developed for health monitoring by integrating optoelectronic devices with similar device structures or fabrication techniques (*29*, *30*). All-organic optoelectronic pulse oximetry has reached commercial levels by combining vacuum-evaporated rigid OLEDs and flexible OPDs (*31*). Flexible large-area OPDs also exhibit low-noise to silicon photodiodes for pulse oximetry (*32*). More comfortable and stable pulse oximetry can be achieved by integrating flexible or ultraflexible organic optoelectronic devices (*33*, *34*). In the aforementioned reports, the wearable sensors were fabricated via vacuum-evaporated processing. More recently, solution-processed OLEDs or OPDs have been developed for optoelectronic sensors to realize scalable production (*35*, *36*).

However, it is challenging to scale up integrated organic optoelectronic wearable sensor manufacturing via all-solution processing, primarily owing to the complex device structure and fabrication process of organic optoelectronic devices. Owing to the tunable energy bandgaps of semiconductors in OPVs, OLEDs, and OPDs, using different device structures with diverse interface layers can result in complex fabrication steps and multiple layers for integrated electronics, diminishing the easy simultaneous fabrication of different devices on the same substrate. Furthermore, the instability of solution-processed interfaces and electrode materials to water and oxygen can result in long-term stability problems in organic optoelectronic devices for application in wearable electronics.

Here, we establish all-solution processes to manufacture a wearable self-powered photoplethysmogram (PPG) sensor. Specifically, we present the development of a facile and universal trilayered all-solution-processed device structure for ultraflexible organic optoelectronics, namely, OPVs, OPDs, and OLEDs, which can be all-coated on ultrathin substrates without interface layers. This is attributed to solution-processed electrodes in ambient air, namely, blade-coated poly(3,4-ethylene dioxythiophene):poly(styrenesulfonate) (PEDOT:PSS) electrode with a high-work function and spray-coated eutectic gallium-indium (EGaIn) electrode with a low-work function that satisfies the requirements of charge transport and collection. The all-solution-processed organic optoelectronic devices exhibit similar performance to that of vacuum-evaporated devices and improved stability in ambient air. The robust interface between sprayed EGaIn and organic semiconductors improves the air stability of the organic optoelectronic device. Last, we successfully integrated all-solution-processed organic optoelectronic devices to realize a self-powered health monitoring electronic system by combining all-solution-processed OLEDs and OPDs, demonstrating the detection of signals after storing for 35 days in air.

## RESULTS

### Device design and materials for all-solution processing

**Fig. 1.**
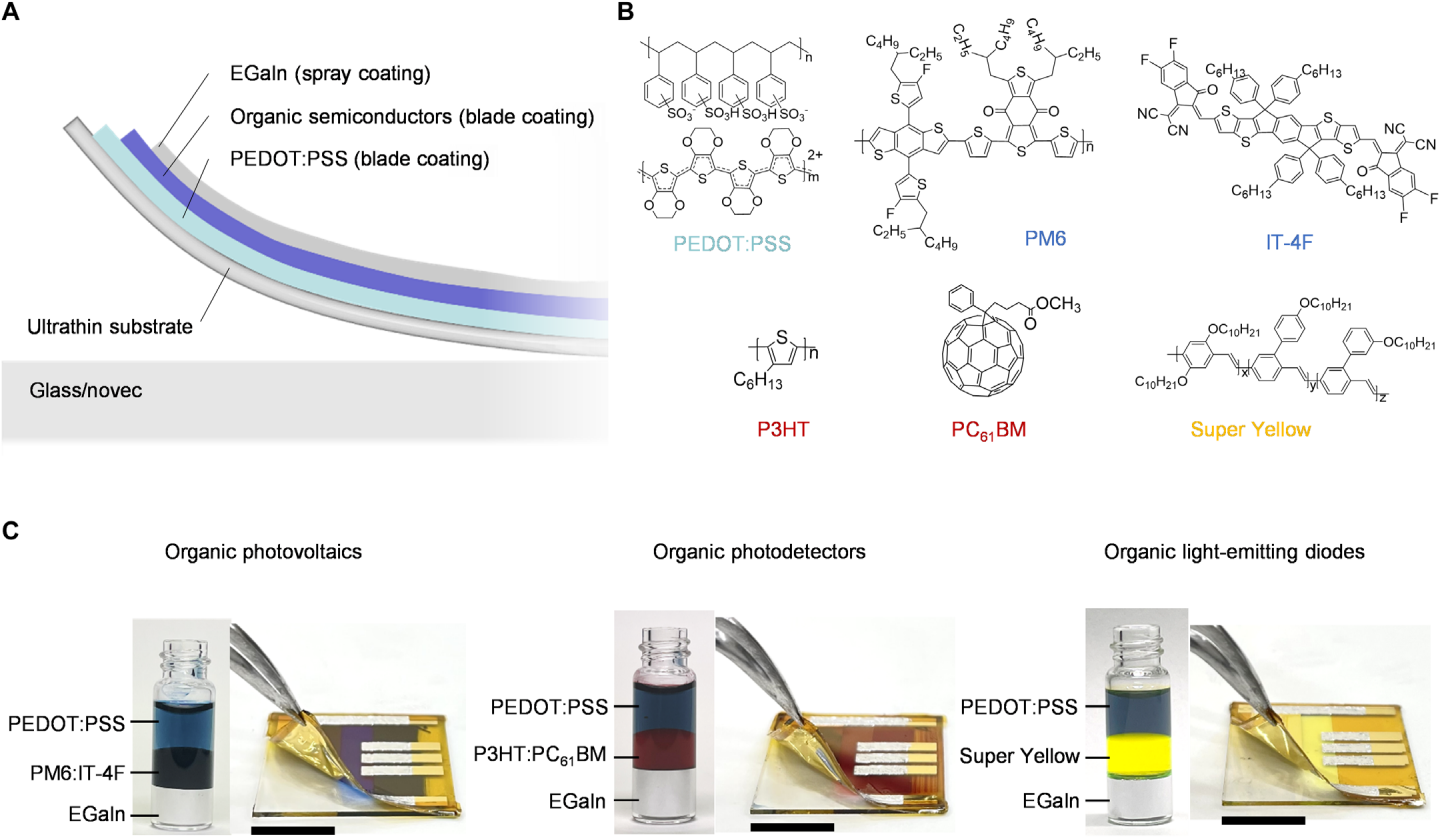
Device structure and materials. (**A**) Cross-sectional diagram of the all-solution-processed organic optoelectronic devices and fabrication process on the ultrathin substrate. (**B**) Chemical structure of the polymer and small-molecule materials used in the all-solution-processed optoelectronic devices. (**C**) Photographs of solutions and delamination process of the ultrathin organic optoelectronic devices from glass substrates. The solution of electrodes and organic semiconductor materials are stored in one vial and separated automatically. Scale bar, 1 cm.

Figure 1A shows the trilayer device structure of the ultraflexible organic optoelectronic devices and the adopted solution-processed techniques. For solution-processed organic optoelectronic devices, the interpenetration between interface layers should be avoided. Our devices were fabricated entirely via solution processing, enabling them to be combined with solution-processed techniques (blade coating and spray coating) for large-scale production. Figure 1B illustrates the chemical structure of the polymer and small molecule used in this study. PEDOT:PSS is used as a bottom transparent electrode. Poly[(2,6-(4,8-bis(5-(2-ethylhexyl-3-fluoro)thiophen-2-yl)-benzo[1,2-b:4,5-b′]dithiophene))-alt-(5,5-(1′,3′-di-2-thienyl-5′,7′-bis(2-ethylhexyl)benzo[1′,2′-c:4′,5′-c′]dithiophene-4,8-dione)] (PM6) and poly[(2,6-(4,8-bis(5-(2-ethylhexyl-3-fluoro)thiophen-2-yl)-benzo[1,2-b:4,5-b′] dithiophene))-alt-(5,5-(1′,3′-di-2-thienyl-5′,7′-bis(2-ethylhexyl)benzo[1′,2′-c:4′,5′-c′] dithiophene-4,8-dione)] (IT-4F) are used as organic active layers in the OPVs. Poly(3-hexylthiophene-2,5-diyl) (P3HT) and [6,6]-phenyl-C61-butyric acid methyl ester (PC_61_BM) are used as organic active layers in the OPDs. OPVs convert a wide solar spectrum using broad-absorbing materials, while OPDs detect specific wavelengths using wavelength-sensitive materials (*5*, *28*, *37*). Super Yellow (SY) is used as an organic active layer in the OLEDs. Figure 1C displays a photograph of the materials solution and fabricated device of the all-solution-processed OPV, OPD, and OLED devices on an ultrathin substrate. Active layer solutions of different types of organic optoelectronics and electrode materials are stored in three vials. Three separate layers are formed between the organic active layer solution, aqueous PEDOT:PSS solution, and the liquid metal (EGaIn) owing to their mutual orthogonality. Upon exposing the solution in air for 30 days (fig. S1), the boundary of each solution remains clear. The orthogonal solution prevented infiltration between two functional layers during deposition and stabilized the interface.

### Preparation of thin and continuous liquid metal film on organic semiconductors

**Fig. 2.**
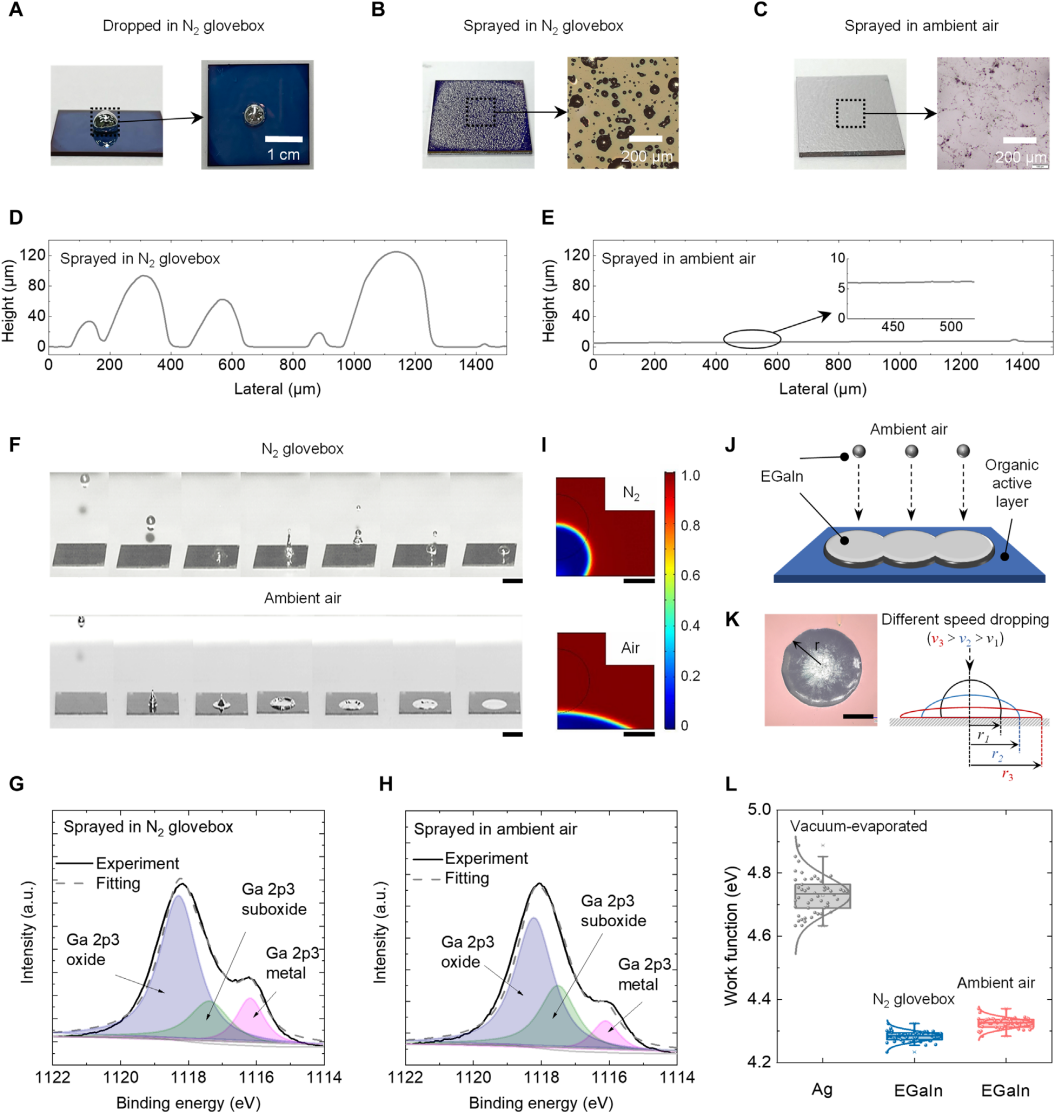
Thin EGaIn film preparation and characterization. Photographs and microscope images for (**A**) dropping EGaIn on PM6:IT-4F active layer in a N_2_ glovebox, (**B**) spraying EGaIn on PM6:IT-4F active layer in a N_2_ glovebox, and (**C**) spraying EGaIn on PM6:IT-4F active layer in ambient air. The thickness distribution of the sprayed EGaIn in (**D**) N_2_ glovebox and (**E**) ambient air. (**F**) Image sequence showing the behavior of a 0.2-g EGaIn droplet dropped in a N_2_ glovebox (top) and ambient air (bottom), captured at a frame rate of 8000 fps. Scale bar, 1 cm. (**G**) XPS spectra of Ga 2p of the sprayed EGaIn in a N_2_ glovebox and (**H**) ambient air. (**I**) Simulation results of the drop behavior of EGaIn droplets in N_2_ and air conditions. Black scale bar, 1 μm. (**J**) Schematic diagram of the continuous dropping of multiple EGaIn droplets. (**K**) Image and schematic diagram of the EGaIn covered area on organic active layer film. Scale bar, 500 mm. (**L**) Work functions of the evaporated Ag and EGaIn electrodes. a.u., arbitrary units.

We confirm that deposition using tiny droplets of EGaIn in ambient air sprayed with a high-pressure N_2_ flow is the key to producing thin and continuous films on organic semiconductors. Solution-processed electrodes should be moderated underneath the fabricated layer to improve device performance (*38*). EGaIn is a liquid metal at room temperature that has high conductivity, which is suitable for stretchable optoelectronics (*39*–*43*). In our previous study, we demonstrated that EGaIn can be dropped onto an organic active layer as the top electrode in a nitrogen (N_2_) glovebox (*44*). Owing to its large surface energy (*45*), EGaIn cannot spread on the surface of the organic active layer (PM6:IT-4F) and patterning into films (Fig. 2A). Unfortunately, the methods for altering EGaIn wetting, such as through acid-base solutions and metal surface modification, are not compatible with the fabrication of organic photoelectronic devices (*46*, *47*). Considering the thickness, patterning, and uniformity as requirements for ultrathin device operation in large-scale production, we chose the spraying technique to scale up EGaIn deposition. However, EGaIn films sprayed on the surface of PM6:IT-4F in a N_2_ glovebox exhibit discontinuity on the surface of the organic active layer films (Fig. 2B). In contrast, the EGaIn film sprayed with N_2_ flow in ambient air is complete and uniform on the surface of the PM6:IT-4F layer (Fig. 2C). The EGaIn film obtained in N_2_ atmosphere exhibits highly undulating aggregation, with a height of over 120 μm (Fig. 2D). Conversely, the EGaIn film sprayed in air is flat and continuous, with a thickness of less than 8 μm (Fig. 2E).

High-speed cameras were used to capture the behavior of EGaIn droplets in different atmospheres. For easy observation, we use large droplets to magnify the tiny droplets during the spraying process (Fig. 2F and movies S1 and S2). In the N_2_ atmosphere, the EGaIn droplets exhibit bouncing on the PM6:IT-4F active layer. However, this bouncing is suppressed in ambient air. The oxide layer on the surface of EGaIn is responsible for the suppressed bouncing. We analyzed the EGaIn films sprayed in different atmospheres via high-resolution x-ray photoelectron spectroscopy (XPS). As shown in (Fig. 2 G and H), the pure Ga metal content (centered at approximately 1116.1 eV) in Ga 2p spectral illustrates differences (*48*). EGaIn sprayed in air shows decreased pure Ga 2p3 metal peak and increased Ga 2p3 oxide and suboxide peaks, indicating that additional gallium oxide is formed in air. Moreover, the O 1s XPS spectra peaks of the EGaIn sprayed in both atmospheres confirm higher oxidation when sprayed in ambient air owing to the high density of the O 1s oxide peak (fig. S2). Notably, EGaIn surfaces are rapidly oxidized when exposed to air, and gallium oxide has an extremely strong surface adhesion (*45*, *49*, *50*). The oxide can continue to form after the liquid metal is subjected to a large yield stress to generate flow, eventually stabilizing the shape (*51*, *52*).

We conducted finite element analysis using COMSOL software to investigate the manner in which the atmosphere could influence the spreading of EGaIn on organic semiconductors during spraying. In the spraying process, the EGaIn was simplified as a tiny droplet with a radius of 1 μm. Different surface tensions were set to simulate surface oxidation caused by air and nitrogen atmospheres. The simulation results show that in the air atmosphere, the EGaIn droplets exhibit suppressed bouncing and rapid spreading, which is similar to the results captured using high-speed cameras (Fig. 2I). The detailed simulation results of EGaIn spreading over time are displayed in fig. S3. Such suppressed bouncing behavior is beneficial for the stacking of EGaIn during the spraying process. Figure 2J illustrates a schematic diagram of the continuous dropping of multiple EGaIn droplets. The actual spreading and stacking of different droplets confirm this benefit from suppressing bouncing (fig. S4). Figure 2K shows the top view images of PM6:IT-4F film covered with EGaIn film. A linear correlation exists between the size (expansion radius: *r*) of the EGaIn film coverage and the energy (impact speed: *v*) of the droplet impacting the surface (fig. S5). The schematic diagram shown in Fig. 2K shows that a large impact speed *v*_3_ will result in a larger expansion radius *r*_3_.

The above results indicate that the preparation of continuous and uniform EGaIn film on an organic active layer can be classified into four stages: (i) atomization stage: the EGaIn is atomized into small droplets by high-pressure N_2_ flow; (ii) injection stage: high-pressure N_2_ gas drives small droplets to be ejected from the nozzle; (iii) impact stage: EGaIn droplets impact the organic semiconductor film and deform; and (iv) accumulation stage: EGaIn droplets are completely spread on the surface of the organic semiconductor film while maintaining the spread shape for accumulation. To further confirm the key to the above stage, we sprayed EGaIn in ambient air using high-pressure air flow. The resulting EGaIn film exhibited additional holes (fig. S6), although the holes are reduced after extending the spraying time and the thickness of the prepared film exceeds 150 μm. This is related to the excessive oxidation and increased viscosity of EGaIn in the atomization stage (*53*). The simulation results shown in fig. S3 indicate that when the viscosity of the EGaIn is increased, the spreading of the droplet is inhibited.

The work functions of EGaIn both dispensed in N_2_ and air are also evaluated (Fig. 2L). The EGaIn dropped in the N_2_ glovebox exhibited a work function of −4.29 eV, and the sprayed EGaIn in ambient air exhibited a work function of −4.33 eV, indicating the oxidized surface of EGaIn in air while ensuring that a good energy band matching is obtained as an ETL for organic semiconducting layers. The appropriate work function and uniform EGaIn film enable the direct deposition of electrodes onto the surface of the organic semiconductor. In summarizing previous large-scale depositions of EGaIn for OPV, our proposed device structure eliminates the need for additional electron interface layers, further simplifying the device structure (table S1).

### All-solution-processed ultrathin organic optoelectronic devices

**Fig. 3.**
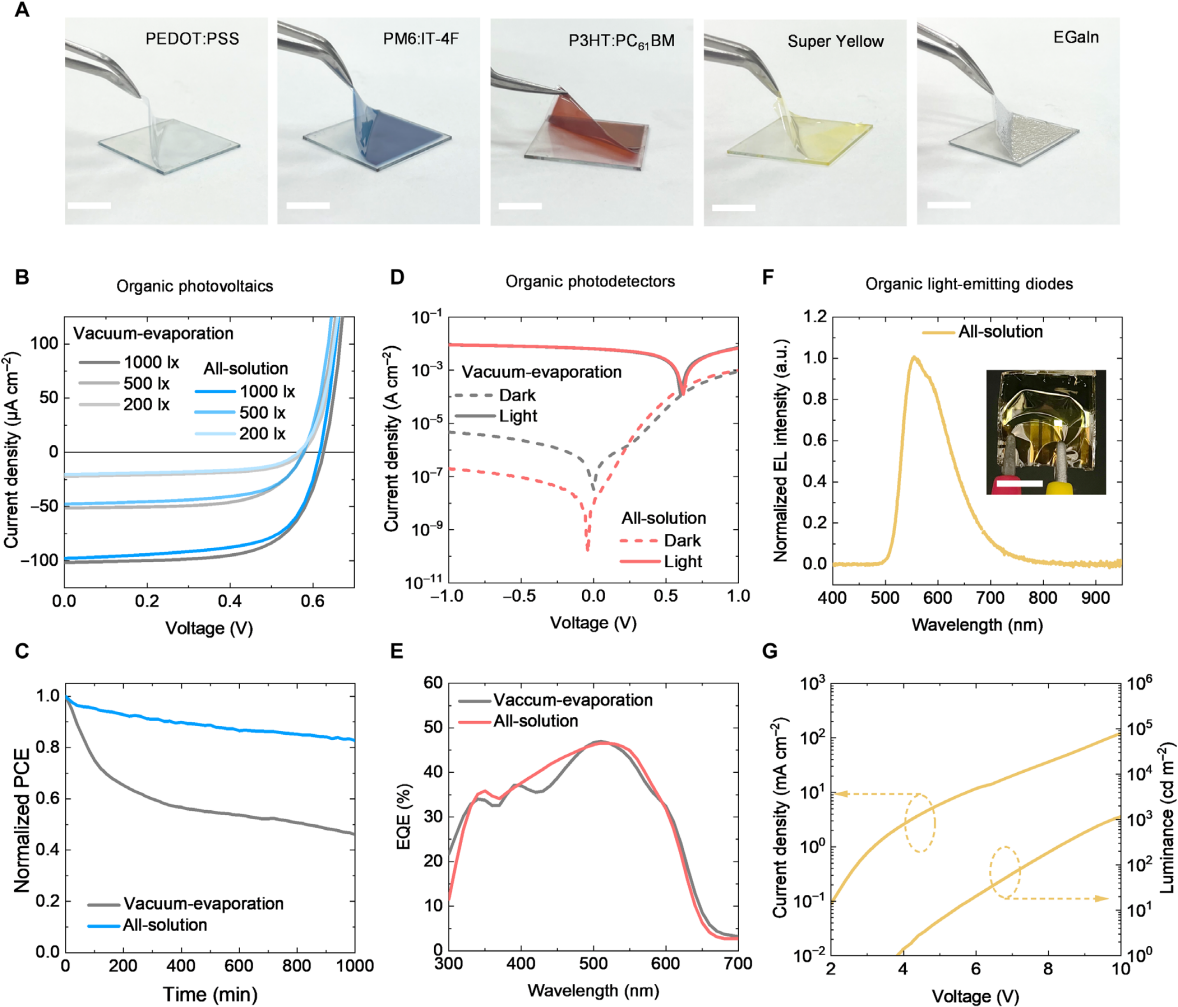
All-solution-processed ultrathin organic optoelectronic device performance. (**A**) Delamination process of blade-coated PEDOT:PSS, blade-coated PM6:IT-4F, blade-coated P3HT:PC_61_BM, blade-coated SY, and spray-coated EGaIn on ultrathin substrate. Scale bar, 1 cm. (**B**) *J*-*V* curves of the all-solution-processed and vacuum-evaporated single-junction OPV under LED light. (**C**) Normalized PCE of the indoor MPP tracking of the all-solution-processed and vacuum-evaporated OPVs in ambient air. (**D**) Dark and light *J*-*V* curves of the all-solution-processed and vacuum-evaporated OPDs. (**E**) EQE spectra of the all-solution-processed and vacuum-evaporated OPDs. (**F**) Normalized electroluminescence (EL) spectra of the all-solution-processed OLEDs. Inset displays a photograph of the ultrathin all-solution-processed OLEDs during operation. Scale bar, 1 cm. (**G**) Current density–luminance–voltage curves of the all-solution-processed OLEDs.

Each functional layer was first coated and optimized on an ultrathin substrate. The ultrathin substrate (parylene/SU-8) exhibited a transmittance of over 90% at 550 nm and sufficient smoothness for optoelectronic substrates (fig. S7). The detailed fabrication process of the ultrathin substrate is shown in fig. S8 and Materials and Methods. The functional layers, including PEDOT:PSS, PM6:IT-4F (for OPVs), P3HT:PC_61_BM (for OPDs), and SY (for OLEDs), can be coated on the ultrathin substrate and peeled from the supporting glass (Fig. 3A). The surface roughness of the film prepared via blade coating is similar to that prepared via spin coating (fig. S9). Different coating speeds can obtain organic functional films with different thicknesses to satisfy the requirements of device fabrication (fig. S10).

A conductive PEDOT:PSS was used as simplified transparent electrodes without an additional HTL. We added 5 wt % ethylene glycol (EG) and 0.5 wt % Nafion to PEDOT:PSS to enhance the charge collection performance of PEDOT:PSS (*54*, *55*). The modified PEDOT:PSS exhibits an improved work function (from −5.0 to −5.4 eV) (fig. S11). The modified PEDOT:PSS exhibits a larger contact angle (98.9°) than pure PEDOT:PSS (22.6°) (fig. S12). The transmittance and surface roughness evaluations reveal that the modified PEDOT:PSS can maintain both good transmittance and surface roughness applicable for the bottom electrodes of the optoelectronic devices (figs. S13 and S14).

Ultrathin OPVs containing all-solution-processed trilayer structures exhibit acceptable efficiency and improved stability than reference OPVs containing evaporated electrodes. For all-solution-processed OPVs, the device structure is PEDOT:PSS/PM6:IT-4F/EGaIn (fig. S15). By processing the cross section of the sample using a cryo-Ar ion milling device and performing high-magnification, high-resolution observation using a scanning electron microscope (SEM), we confirmed the sequential deposition state of the device functional layers and their good interfacial contact (fig. S16). For comparison, the vacuum-evaporation device with the indium tin oxide (ITO)/PEDOT:PSS/PM6:IT-4F/PDINN/Ag structure is used as a reference. The energy levels of the all-solution and reference devices are displayed in fig. S17. The all-solution-processed OPVs exhibit comparable performance under 1000-lx LED light with a *P*_max_ of 39.38 μW cm^−2^ to the reference OPVs with *P*_max_ of 41.22 μW cm^−2^ (Fig. 3B and table S2). In addition, we fabricated the all-solution-processed OPVs with air flow via air-spraying, and the device shows a much lower *P*_max_ of 21.52 μW cm^−2^ than references (fig. S18). Unencapsulated all-solution-processed devices maintained over 80% of the initial performance after 1000-min maximum power point (MPP) tracking under the LED light. This is remarkably better than the achievement of the reference OPVs (maintaining 46.12% of the initial performance) (Fig. 3C). Flexibility performance is important for wearable electronics. The devices exhibit better flexibility after 500 bending cycles at a bending radius of 5 mm than reference OPVs (fig. S19A). This can be attributed to the unchanged electrical performance of solution-processed electrodes (fig. S19B). Upon attaching the device to the human skin to test its bending performance, following 100 cycles of up to a maximum of 70° bending at the wrist, the device still maintained over 90% of its initial performance (fig. S19, C and D). The all-solution-processed OPVs show a performance of power conversion efficiency (PCE) = 9.96% (*V*_OC_ = 0.81 V, fill factor = 0.65, and *J*_SC_ = 18.86 mA cm^−2^) under AM1.5G 1 sun (fig. S20). The devices exhibit similar performances as the vacuum-evaporated reference, and the slight difference in current density is caused by bottom electrode transmission. The stability of the all-solution-processed OPVs under 1-sun light was also tested (fig. S21), confirming better operational stability in both outdoor and indoor light conditions than reference OPVs containing evaporated electrodes.

The improved air stability is caused by the robust interface between organic semiconductors and sprayed EGaIn. To demonstrate this result, the active layer (PM6:IT-4F) film with the sprayed-coated EGaIn electrode was treated with oxygen plasma followed by the removal of the EGaIn film. Following plasma treatment, the absorption of the organic active layer film showed no decrease with the protection of the EGaIn film (fig. S22). Subsequently, the electron-only device with the EGaIn electrode (ITO/PEI-Zn/PM6:IT-4F/EGaIn) was immersed in deionized water for 1 min (fig. S23). Subsequently, the reference electrode PDINN/Ag was easily peeled, and the current density was decreased. However, the device with the sprayed EGaIn electrode exhibits no change in current density. These characteristics demonstrate the excellent water and oxygen barrier properties of EGaIn electrodes (*40*).

Similar to OPVs, ultrathin OPDs having all-solution-processed trilayer structures exhibit reasonable performance and improved stability. For all-solution-processed OPDs, the device structure was PEDOT:PSS/P3HT:PC_61_BM/EGaIn. A stack of ITO/PEDOT:PSS/P3HT:PC_61_BM/PEIE/Ag was selected as a vacuum-evaporated reference. The performance of the *J-V* curve of the OPDs was first measured (Fig. 3D). The all-solution-processed OPDs have a lower dark current density than that of the vacuum-evaporated reference. Figure S24, which counts the dark current distribution, further illustrates this result. Figure 3E shows the external quantum efficiency (EQE) spectra of the OPDs, and both all-solution-processed and vacuum-evaporated references exhibit a high EQE of 50% at 550 nm. The light intensity dependence of the all-solution-processed OPDs was also evaluated by changing the filter under a 1-sun light (fig. S25). The all-solution-processed OPDs demonstrate a fitting curve closer to 1 than the vacuum-evaporated devices (fig. S26). More detailed characterization, including responsivity, detectivity, and response time, is evaluated for comparison with the vacuum-evaporated reference (fig. S27). The detailed performances are listed in table S3. The all-solution-processed OPDs exhibit excellent storage stability under dark ambient air without encapsulation. The dark current density of the all-solution-processed OPDs indicates no change after 1000 min of storage in ambient air (fig. S28). The all-solution-processed OPDs maintained basic light properties after storage in ambient air, whereas vacuum-evaporated reference lost their fundamental ability (fig. S29). Even after 35 days in the air, the dark current density remains close to the initial value (fig. S30).

Ultrathin OLEDs were also fabricated using the trilayer all-solution-processed device structure to further demonstrate universality. For the all-solution-processed OLEDs, the device structure was PEDOT:PSS/SY/EGaIn. An SY polymer was used as the emission layer. Figure 3F illustrates the electroluminescence spectra of the devices. The inset shows the freestanding ultraflexible all-solution-processed OLED device peeling from the glass substrate. The current density–luminance–voltage curves of the all-solution-processed OLEDs show its turn-on voltage as 3.8 V (Fig. 3G). The all-solution-processed OLEDs on ultraflexible substrates show comparable values after peeling from a glass substrate (fig. S31). The vacuum-evaporated device with the ITO/PEDOT:PSS/SY/PEIE/Ag structure was also evaluated as a reference (fig. S32). The performance is summarized in table S4. All-solution-processed methods impart OLEDs with great designability, and ultrathin all-solution-processed OLED displays with complex shapes can be obtained (fig. S33) by spraying top EGaIn electrodes of different masks. To verify the reliability of large-scale production of this trilayer all-solution structure, we reproduced the organic optoelectronic devices (OPVs, OPDs, and OLEDs) and statistically calculated their performance distribution (fig. S34); good repeatability is beneficial to the scale-up production of all-solution-processed devices.

### Large-area production and integrated sensor application

**Fig. 4.**
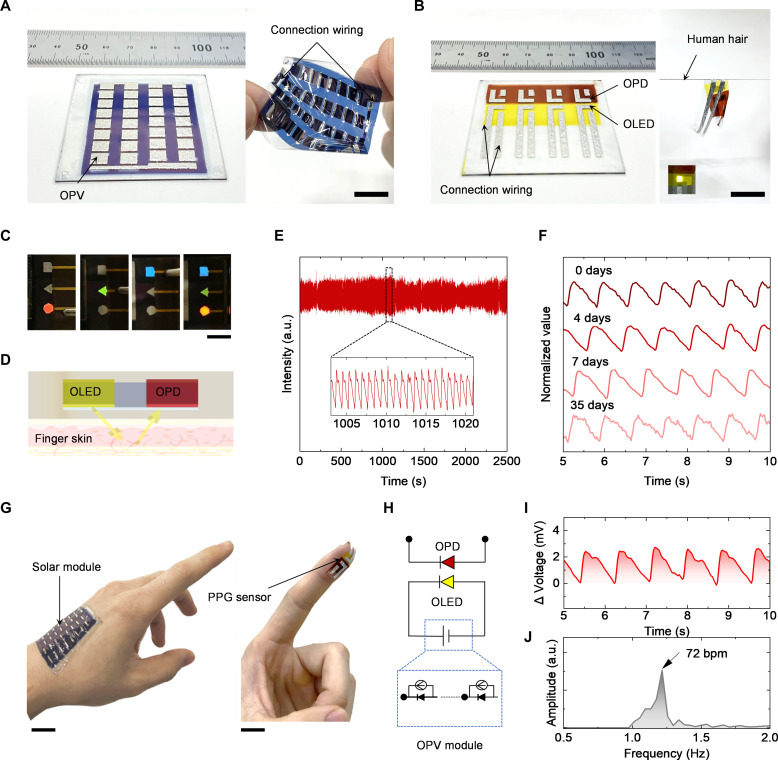
All-solution-processed organic optoelectronic applications. (**A**) Photograph of a fully coated large-area solar module on an ultrathin substrate and after peeling from a glass substrate support. Scale bar, 1 cm. (**B**) Photograph of a fully coated PPG sensor based on OLEDs and OPDs on ultrathin substrate and after peeling from glass substrate support. The inset displays OLEDs operating on 10 V. Scale bar, 1 cm. (**C**) All-solution-processed multicolor displays of different shapes. Scale bar, 1 cm. (**D**) Schematic describing the operational mechanism of PPG by OLEDs and OPDs. (**E**) Long-term measurement of the pulse wave of the all-solution-processed PPG sensor. (**F**) Air stability of the PPG signal of all-solution-processed electronic systems. Devices were stored in the dark under ambient air for days. (**G**) Photograph of a large-area, all-solution-processed solar module attached on a hand and a PPG sensor on a finger. Scale bar, 1 cm. (**H**) Electrical circuit of a self-powered PPG sensor. (**I**) Output voltage characteristics of all-solution-processed OPDs with PPG measurement. Large-area all-solution-processed OPV modules powered the all-solution-processed OLEDs with 1-sun illumination from a solar simulator. (**J**) Blood pulse frequency is 72 bpm from the measurement results.

We demonstrated the applicability of simple all-solution processing to large-area fabrication of ultraflexible organic optoelectronics systems. We designed a large-area OPV module and PPG sensor array by integrating OLEDs and OPDs on 5 cm–by–5 cm ultrathin substrates (fig. S35). Organic polymer films and EGaIn electrodes were deposited on large substrates, confirming that the fabrication of universal trilayer devices can be applied to large-area devices (figs. S36 and S37). Photographs of a fully coated large-area solar module and PPG array based on OLEDs and OPDs on ultrathin substrates are displayed in (Fig. 4 A and B, respectively). The peeling process of the sensor is shown in fig. S38. Moreover, we simultaneously prepared multicolor displays of different shapes through this trilayer structure, thus demonstrating the applicability and designability of this all-solution processing (Fig. 4C). The detailed design and device photograph are shown in fig. S39.

The operation mechanism of a PPG sensor and long-term pulse wave measurement are shown in (Fig. 4 D and E, respectively). The all-solution-processed sensor showed typical PPG signals and maintained signals related to the blood pulse wave even after long-term (over 35 days) exposure in ambient air (Fig. 4F), and the initial signal-to-noise ratio (SNR) of all-solution-processed sensor achieved 23 dB. The excellent stability of the all-solution-processed devices with universal trilayer structures enabled such long-term detection of the sensor. The detected signals were compared with commercial PPG sensors and achieved 0.2% error for the pulse rate (fig. S40). Because of the good flexibility and skin conformability, the device can achieve high SNR with minimized light dissipation and motion artifacts than rigid commercial device (*56*). We have also conducted the flexibility test for the sensor. The flexibility of all-solution-processed sensor was evaluated at different bending radius, and the device still maintained over 80% of the initial SNR value. This is due to the stable performance of OPD and OLED during bending (fig. S41). We operated the OLEDs in a PPG sensor with different driving voltages, and when the driving voltage of OLEDs in the PPG sensor was changed, the all-solution-processed sensor could generate a stable signal value at a low driving voltage of OLEDs (fig. S42).

It is crucial to ensure that the output current and voltage of the OPV modules are equal to that of the OLED for efficient detection to achieve sufficient output power from the OPV to drive the OLED in the PPG sensor for self-powered systems (fig. S43) (*33*). By changing the number of series cells of the OPV module, the different power curves obtained are compared with the working power of the OLED in PPG sensors, and it was found that the maximum power output point of the 16-subcell is closest to the working power of the OLED (fig. S44A). The large-area solar module can produce a maximum output power of 25.87 mW at *V*_max_ = 8.55 V and *J*_max_ = 3.05 mA under 1 sun (fig. S44B), ensuring sufficient current and voltage to drive the OLEDs in the PPG sensor. Since these all-solution-processed devices are sufficiently thin, the large-area solar module and PPG sensor can be attached to the human skin (Fig. 4G). The cytotoxicity test on the device verified its good biocompatibility and low toxicity (fig. S45) (*57*–*59*). Last, we integrated the solar module with the PPG sensor (Fig. 4H). The all-solution-processed PPG sensor driven by a solar cell operates successfully (Fig. 4I), showing 72 beats per minute (bpm) from the measurement results (Fig. 4J). The detailed signal processing and setup are shown in Materials and Methods and fig. S46. These results represent the applicability of the proposed all-solution-processed organic optoelectronic devices with universal trilayer structures. To emphasize the processability of our proposed device structure, we summarized the recently reported PPG sensors based on OPDs and OLEDs (table S5) with the fabrication process, substrate thickness, SNR, and power source. Compared with previously reported solution-processed organic and perovskite optoelectronic devices, the proposed trilayer structure can be prepared using blading and spraying techniques, enabling rapid fabrication of large-area optoelectronic devices while maintaining complex device compositions. In the future, we will further improve the permeability and signal processing of the device to enhance the comfort and motion artifact recognition (*59*–*63*). These solution-processing techniques can collectively promote the commercial production of organic and perovskite semiconductor devices.

## DISCUSSION

In this study, we demonstrated a trilayer structure for all-solution-processed organic optoelectronic devices on an ultrathin substrate. The solution-processed structure is simple and stable while retaining the flexibility of the ultrathin device. Through this structure, large-scale production of OPVs, OLEDs, and OPDs could be simultaneously fabricated on an ultrathin substrate. This provides a promising avenue to realize the high-throughput production of ultrathin electronic devices integrating complex functions.

## MATERIALS AND METHODS

### Materials

PEDOT:PSS (Clevious PVP Al4083) and PEDOT:PSS (Clevious PH1000) were purchased from Heraeus. The PDINN solution (1 mg/ml, 1-Material) was prepared by dissolving amine-functionalized perylene-diimide (PDINN) in methanol (CH_3_OH, FUJIFILM Wako Chemicals). A fluorinated polymer-releasing layer was prepared by mixing Novec 1700 (3M Company) with Novec 7100 (3M Company) at a 1:8 ratio. EGaIn, a liquid metal alloy, was prepared by melting mixed-metal pieces of gallium (99.99%, Furuuchi Chemical) and indium (99.99%, Furuuchi Chemical) at a 3:1 weight ratio at 100°C for 3 hours in nitrogen atmosphere. A glass substrate with a patterned 150-nm-thick ITO electrode was purchased from GEOMATEC Co. Ltd. PM6 and IT-4F were purchased from 1-Material. A PM6:IT-4F solution (20 mg/ml) was prepared by blending PM6 (1-Material) and IT-4F (1-Material) at a 1:1 weight ratio in a chlorobenzene solution (CB, FUJIFILM Wako Chemicals) with 0.5% 1,8-diiodooctane (DIO) additive (Sigma-Aldrich). A yellow light-emitting polymer, SY, was purchased from Sigma-Aldrich. P3HT was purchased from Rieke Metals LLC. PC_61_BM was purchased from Solenne BV. Anhydrous acetone, isopropyl alcohol (IPA), anhydrous ethanol, 2-methoxyethanol, chloroform, and chlorobenzene were purchased from Wako Pure Chemical Ltd. PEIE (80% ethoxylated solution, ~40 wt % in water) and Nafion (containing 34% water) were purchased from Sigma-Aldrich.

### Device fabrication

For the ultrathin substrate fabrication, the glass substrates were sequentially cleaned using deionized water, acetone, and IPA for 15 min via ultrasonic treatment. Subsequently, oxygen plasma treatment was conducted for 10 min to remove residual chemicals on top of the glass surface. A fluorinated polymer layer (Novec 1700, 3M) was spin-coated onto a cleaned glass substrate for 60 s at 2000 rpm, and a 1.5-μm-thick parylene film was deposited via chemical vapor deposition onto a glass plate with the surface coated in the fluorinated polymer layer. Following deposition of the parylene layer, a 500-nm-thick epoxy (SU-8 3005, MicroChem) layer was spin-coated (5000 rpm for 60 s) to obtain planarization. The film was annealed at 95°C for 3 min after ultraviolet (UV) exposure and then annealed in a N_2_ atmosphere at 180°C for 30 min.

For vacuum-evaporated ultrathin reference OPVs and OPDs, the device structures are ITO/PEDOT:PSS/PM6:IT-4F/PDINN/Ag and ITO/PEDOT:PSS/P3HT:PC_61_BM/PEIE/Ag, respectively. First, Cr/Au contact pads (3.5-nm-thick Cr and 100-nm-thick Au) were deposited onto the ultrathin ITO electrodes via vacuum deposition. Subsequently, the sample was treated with oxygen plasma for 1 min. A 35-nm-thick PEDOT:PSS layer was used as the HTL. The substrates were spin-coated with a PEDOT:PSS (Clevious PVP Al4083) (3500 rpm for 30 s) solution. The substrates were then baked in air at 150°C for 10 min. Upon annealing the substrates, they were transferred to a N_2_ glovebox. For OPVs, the PM6:IT-4F solution (1:1, 20 mg/ml, 0.5 wt % DIO, in clobenzene) was deposited onto the as-prepared PEDOT:PSS surface by spin coating at 1500 rpm for 40 s, followed by annealing at 100°C on a hot plate for 10 min. The prepared PDINN solution was then spin-coated onto the annealed PM6:IT-4F layer at 5000 rpm for 40 s. For OPDs, the P3HT:PC_61_BM solution (1:1, 40 mg/ml, in clobenzene) was deposited onto the as-prepared PEDOT:PSS surface by spin coating at 600 rpm for 90 s, followed by annealing at 150°C on a hot plate for 10 min. The prepared PEIE solution was then spin-coated onto the annealed P3HT:PC_61_BM layer at 5000 rpm for 40 s. Following the deposition of the organic active layer, the Ag (100 nm) cathode was deposited through a shadow mask in a vacuum evaporator (EX-200, ULVAC).

For the fabrication of the all-solution-processed OPVs, OLEDs, and OPDs, the modified PEDOT:PSS was first coated onto the ultrathin substrates using a glass substrate support with a thickness of 100 nm, followed by annealing at 150°C on a hot plate for 10 min. The modified PEDOT:PSS solution was prepared by adding 5 wt % EG and 0.5 wt % Nafion into PEDOT:PSS PH1000 and stirring for 5 hours. Subsequently, PM6:IT-4F, SY, and P3HT:PC_61_BM were deposited on the modified PEDOT:PSS via blade coating. Following the deposition of the organic active layer, the substrates were transferred to a N_2_ glovebox for post-annealing. Last, EGaIn was sprayed on the surface of the active layer with the shadow mask. To prevent excessive oxidation of EGaIn, it was stored in a N_2_ glovebox before spraying. An airbrush with 0.3 μm of a caliber was connected to high-pure nitrogen. The pressure of the nitrogen gas source was 0.5 MPa. The distance between the airbrush and substrate was 20 cm. To prevent dispersion of the liquid metal, the substrate was placed in a box with a depth of 20 cm. The shadow mask was tightly clamped to the substrate. The spraying process lasted less than 5 s to form a continuous film.

For all-solution-processed organic integrated electronic fabrication, a large-area, all-solution-processed OPV solar module was obtained by connecting subcells in parallel and series. First, the modified PEDOT:PSS was coated on a large-area (5 cm by 5 cm) ultrathin substrate. The PEDOT:PSS was then patterned using oxygen plasma with a shadow mask. The etched area was treated with oxygen plasma (PC-300, SAMCO) at 10 Pa, 5 sccm, and 100 W for 1 min. The effective PEDOT:PSS area was protected by the shadow mask. After patterning the PEDOT:PSS, the PM6:IT-4F active layer was coated on the top of the modified PEDOT:PSS. Subsequently, part of the active layer was removed to expose the bottom PEDOT:PSS to facilitate parallel and series connections between the subcells. EGaIn electrodes were deposited on the top of the active layer with a shadow mask. Each subcell had an area of 0.1 cm^2^, and the total effective area was 3.2 cm^2^. The all-solution-processed PPG sensor fabrication process was similar to the large-area solar module. Upon patterning PEDOT:PSS, first, P3HT:PC_61_BM was prepared on the modified PEDOT:PSS and the heat treatment was completed. SY was coated on the ultrathin substrates to prevent the degradation of the OLED performance owing to high-temperature treatment from P3HT:PC_61_BM. EGaIn electrodes were deposited on the top of the active layer with a shadow mask. Once the device was fabricated, 1-μm-thick parylene was deposited on the top of the device to prevent the flow of EGaIn.

### Characterization

For the indoor performance test, all the fabricated devices were characterized under a white LED (BLD-100, Bunkoukeiki) at various light intensities. Three different intensities of LED lights (1000, 500, and 200 lx) were used in the analysis, and their corresponding intensities were 311.94, 155.24, and 59.82 μW cm^−2^, respectively. The emission spectra and light intensity of the LED light source were measured using a spectrometer (SCR-300, Bunkoukeiki), and the illuminance was measured using a luxmeter (BK-19S, Bunkoukeiki). For the outdoor performance test, the fabricated devices were characterized under simulated solar illumination (AM 1.5G spectra, 100 mW cm^−2^ calibrated with a standard silicon reference diode). The *J-V* characteristics were recorded using a Keithley 2400 source meter at a rate of 0.2 V s^−1^ in an ambient atmosphere. The light intensity was adjusted using neutral density filters and then calibrated using a reference silicon diode. UV-visible absorption spectra and optical transmittance spectra were acquired using a UV-visible/near-infrared spectrophotometer (V-780, JASCO) in the wavelength range from 300 to 1000 nm. Atomic force microscopic images were obtained using a Shimadzu SPM-9700HT scanning probe microscope in phase mode. The work function of EGaIn was measured using a scanning Kelvin probe (KP020, KP Technology). XPS characterization was performed using a Thermo Fisher Scientific K-alpha instrument with an Al Kα source. The MPP tracking tests were executed under a 1000-lx white LED and AM 1.5G 1 sun. The current density–voltage curves were drawn every 10 min in the forward direction, and the voltage at the MPP was updated accordingly. The measurements were performed in the ambient air condition. Low-resolution cross-sectional SEM images were captured (VHX-D500, Keyence, Japan). The cross-sectional processing device used for high-resolution cross-sectional observation was a cryo-Ar ion milling device (TIC 3X, Leica, Germany), and Ar beam processing was performed at −40°C. High-resolution cross-sectional SEM and energy-dispersive x-ray (EDX) images were obtained using SEM (Quattro S, FEI Company, USA) and EDX (Thermo Fisher Scientific).

The all-solution-processed pulse wave sensor was attached to the finger to measure the output voltage of the OPDs. Different voltages were applied to the OLEDs. The measurements were performed using a semiconductor parameter analyzer (B1500A, Keysight). The instruments were directly connected to the OPDs. The output signal was filtered with a bandpass within the range of 1 to 100 Hz to remove noise from the environment. The SNR was calculated from the signal via Fourier transform of the time-PPG value (*64*). The study protocol was comprehensively reviewed and approved by the ethical committee of the University of Tokyo (approval number, KE20-111).

### COMSOL simulation

Finite element numerical simulation was performed using COMSOL Multiphysics. The time evolution of two-phase laminar flow was simulated in the phase-field method with an axisymmetric model. The initial condition had a spherical droplet of EGaIn with a radius of 1 μm at a distance of 0.5 μm from the substrate. The initial velocity was set to 15 m/s. Densities were 6440 kg m^−3^ for EGaIn and 1.29 kg m^−3^ for atmosphere. Kinetic viscosities were 0.0028 Pa·s for EGaIn sprayed in N_2_ or sprayed in air with N_2_ flow, 0.025 Pa·s for EGain sprayed in air with air flow, and 17.9 × 10^−6^ Pa·s for atmosphere. Surface tensions and contact angles were 625 mN m^−1^ and π/6 for EGaIn sprayed in N_2_, and 356 mN m^−1^ and 2π/3 for EGaIn sprayed in air with N_2_ or air flow. The overall system size was 3 μm in height and radius. The mobility tuning parameter χ was set to 1 kg m s^−1^.

### Cytotoxicity test in vitro

The embryonic mouse fibroblast cell line (NIH3T3) was purchased from the Japanese Collection of Research Bioresources Cell Bank. NIH3T3 fibroblasts were cultured in Dulbecco’s modified Eagle’s medium (DMEM; FUJIFILM Wako Pure Chemical Industries Ltd., Osaka, Japan) supplemented with 10% fetal bovine serum (MP Biomedicals LLC., Illkirch, France), penicillin (1.0 × 10^5^ U/liter), and streptomycin (100 mg/liter) (Nacalai Tesque Inc., Kyoto, Japan) at 37°C with 5% CO_2_. The cells were harvested after reaching 90% of confluence. The cytotoxicity of the device was evaluated by cytotoxicity kit assay and Live/Dead staining with NIH3T3 fibroblast, respectively. According to the standard protocol in ISO 10993-5:2009, the extract solution was obtained by immersing the device (1.5 cm by 1.5 cm) in 1 ml of fresh DMEM for 24 hours. NIH3T3 fibroblasts were seeded in 96-well plates with a density of 5 × 10^4^ cells/ml. The culture medium was replaced with extracts and fresh DMEM as control after 24 hours. After 48 hours of culture, cell viability was determined by Cell Counting Kit-8 (Dojindo, Kumamoto, Japan) according to the manufacturer’s protocol. The absorbance of the medium was measured at 450 nm by a multimode microplate reader (PerkinElmer, Waltham, MA, USA). The viability of cells was visualized by the LIVE/DEAD Cell Imaging Kit (Thermo Fisher Scientific, USA) according to the manufacturer’s protocol. The microscopy images were captured by a fluorescent microscope (Olympus IX71).

## Supplementary Materials

### This PDF file includes:

Figs. S1 to S46 Tables S1 to S5 Legends for movies S1 and S2 References
