# Supplementary material for: All-solution-processed ultraflexible wearable sensor enabled with universal trilayer structure for organic optoelectronic devices
Source: Sci Adv. 2024 Apr 10;10(15):eadk9460. doi: 10.1126/sciadv.adk9460 (PMC11006222; doi:10.1126/sciadv.adk9460)
Supplement: Supplementary file 1 — Figs. S1 to S46 Tables S1 to S5 Legends for movies S1 and S2 References [file sciadv.adk9460_sm.pdf]

Science Advances
AAAS

Supplementary Materials for

All-solution–processed ultraflexible wearable sensor enabled with universal trilayer structure for organic optoelectronic devices

Lulu Sun *et al.*

Corresponding author: Takao Someya, takao.someya@riken.jp; Kenjiro Fukuda, kenjiro.fukuda@riken.jp

Sci. Adv. **10**, eadk9460 (2024)
DOI: 10.1126/sciadv.adk9460

The PDF file includes:

Figs. S1 to S46
Tables S1 to S5
Legends for movies S1 and S2
References

Other Supplementary Material for this manuscript includes the following:

Movies S1 and S2

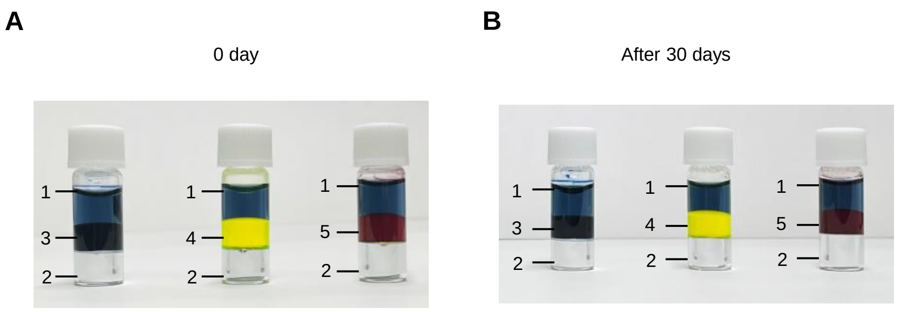

1. PEDOT:PSS, 2. EGaIn, 3. PM6:IT-4F, 4. Super yellow, 5. P3HT:PC_{61}BM.

Fig. S1. Photographs of the functional solution. (**A**) Photograph of the solution used in all-solution processed organic optoelectronic devices. Detailed information on the solution is as follows: 1. PEDOT:PSS, 2. EGaIn, 3. PM6:IT-4F, 4. Super Yellow, and 5. P3HT:PC_{61}BM. (**B**) Solutions were exposed to air for 30 d. Owing to the orthogonality of the solvents, the solution shows a noticeable interface, which could still be observed after 30 d of exposure. The black scale bar indicates 1 cm.

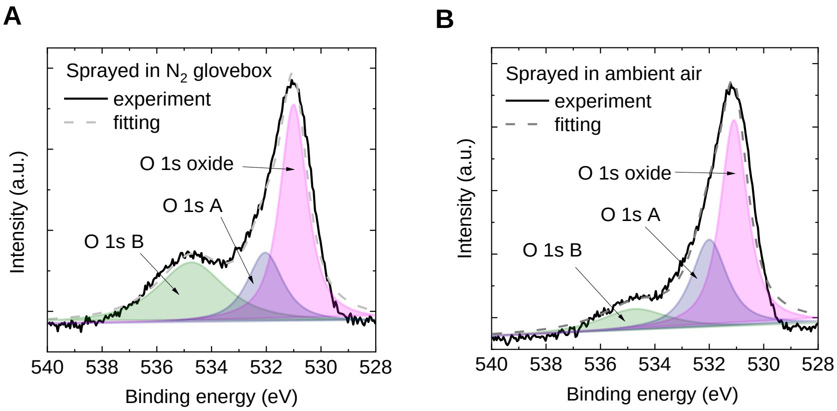

Fig. S2. XPS of the EGaIn. (**A**, **B**) XPS spectra of O 1s of the sprayed EGaIn in (**A**) N_2 glovebox and (**B**) ambient air. O 1s region of the spectrum depicts a minimum of three oxygen species. First peak (O 1s oxide) can be attributed to the inorganic oxides of gallium and indium. The remaining two peaks at higher binding energies (O 1s A and O 1s B) are likely produced by hydroxyl groups and organic oxygen.

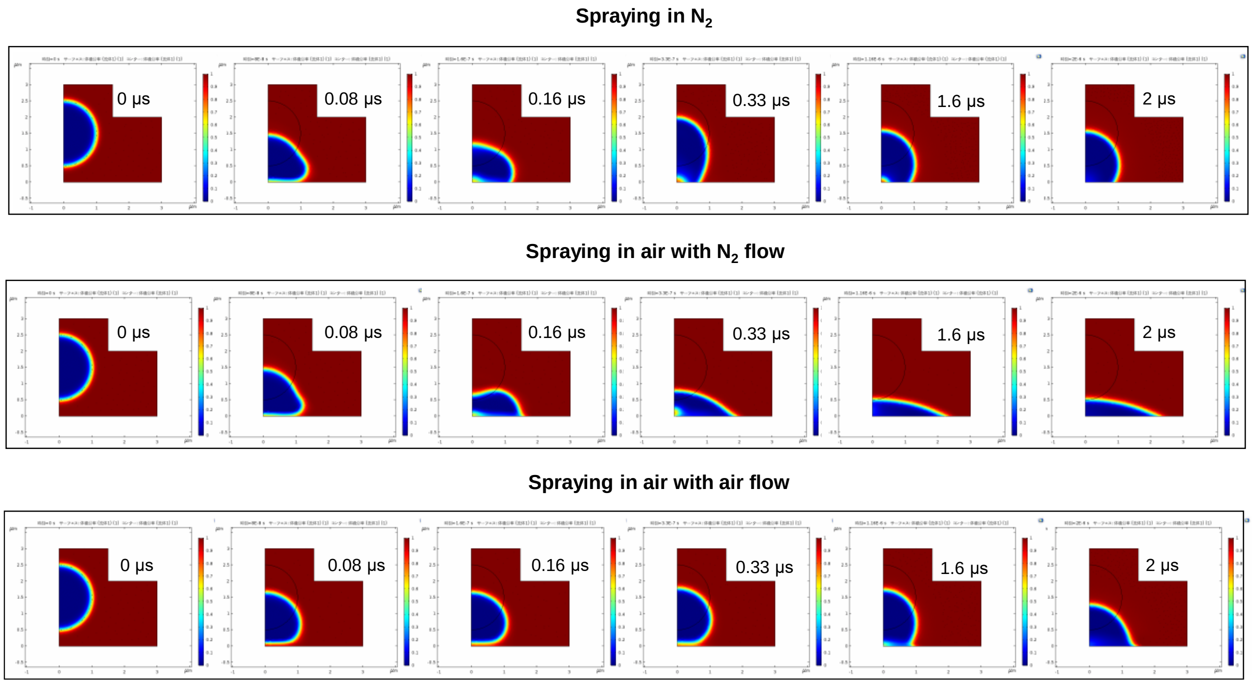

Fig. S3. Simulation of EGaIn droplets with different spraying conditions. The radius of the EGaIn droplets was set to 1 μm to simulate the size of the droplets after atomization during the spraying process. Different viscosities and surface tensions are set for simulating different spray conditions. Detailed parameters are listed in the Materials and Methods section.

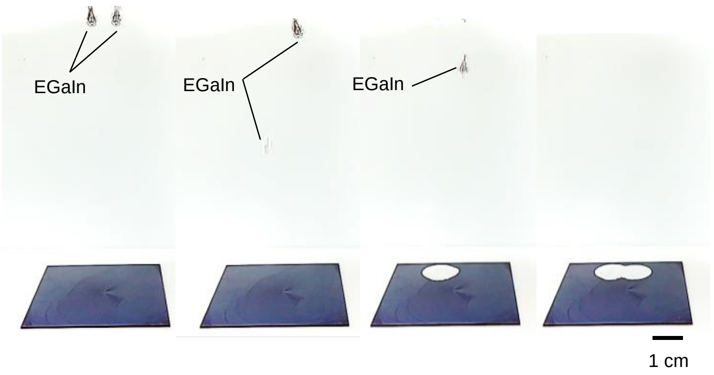

Fig. S4. Multiple droplet stacks. Images of multiple drops of liquid metal falling. The substrate is glass covered with PM6:IT-4F active layer film. The substrate size is 5 cm × 5 cm.

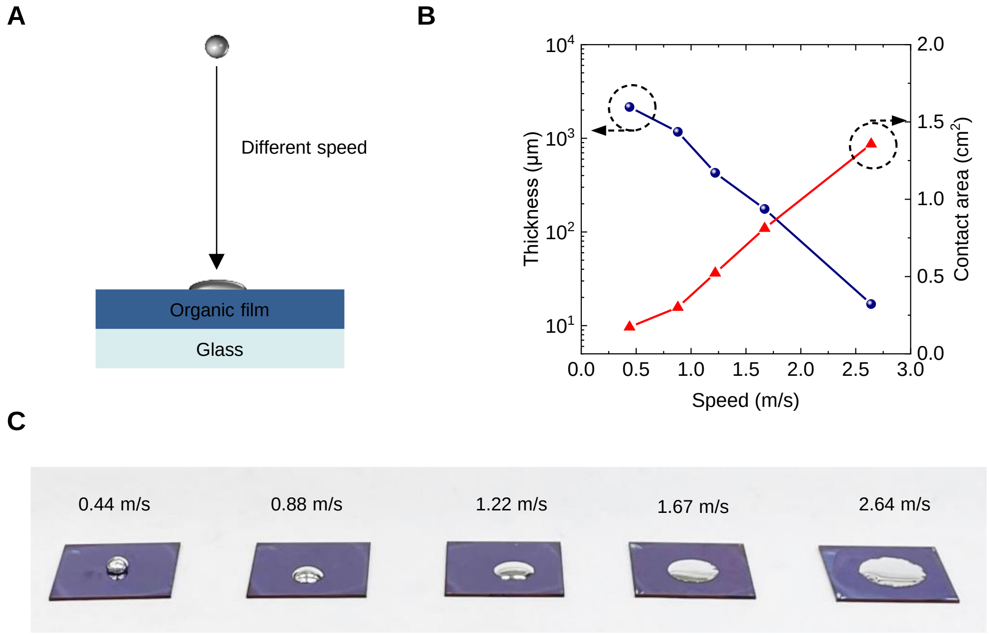

Fig. S5. Relationship between film formation and droplet velocity. (**A**) Diagram of liquid metal droplets falling at different rates. (**B**) Contact area and thickness of EGaIn on the surface of the organic active layer with different drop speeds. (**C**) Photographs of EGaIn dropped on the organic active layer at different speeds. Substrate is PM6:IT-4F/glass, and the substrate size is 2.4 × 2.4 cm.

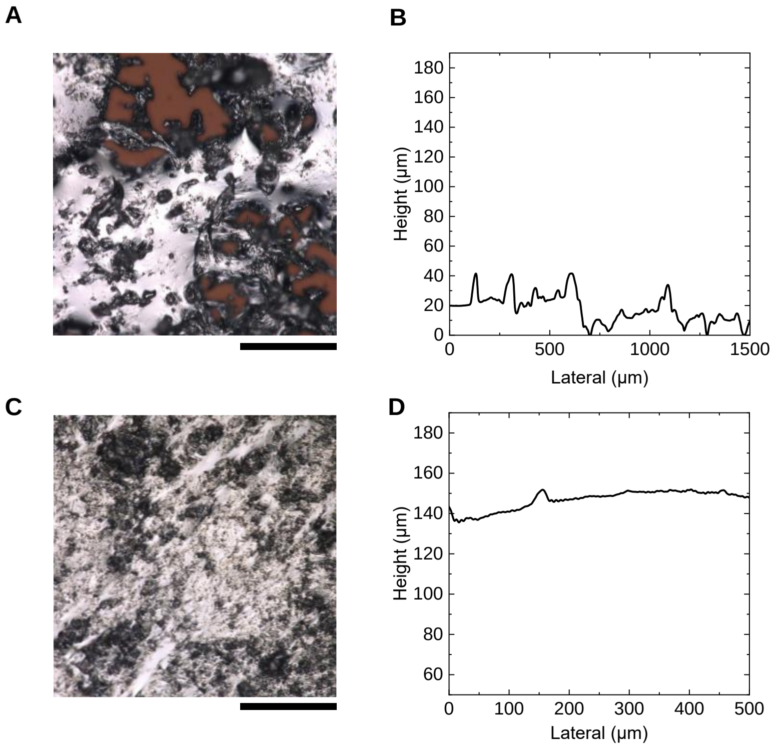

Fig. S6. Spray coating EGaIn with air gas flow. (**A**) Microscope image of the EGaIn film surface. EGaIn was sprayed using high-pressure air flow, the spraying time is 10 s. The black scale bar of each image indicates 100 µm. (**B**) Thickness distribution of EGaIn films sprayed with air flow. Using air as a pressure source for spraying can result in additional pores or holes. (**C**) Microscope image of the EGaIn film surface after extending the spraying time to 30 s for reducing the holes. The black scale bar of each image indicates 100 µm. (**D**) Thickness distribution of EGaIn films sprayed with air flow after extending the spraying time.

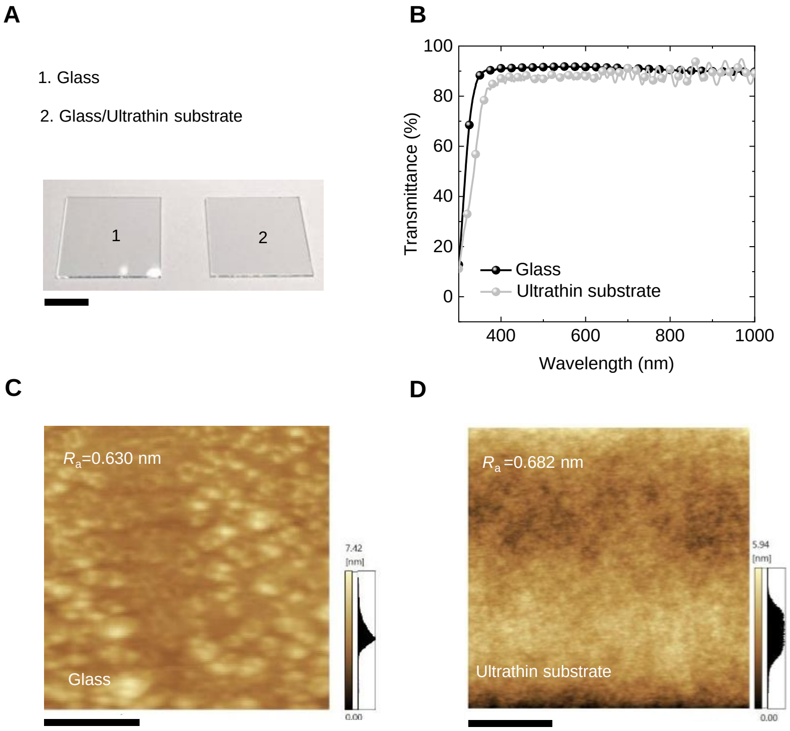

Fig. S7. Transmittance and surface roughness of glass and ultrathin substrate. (**A**) Photograph of the glass substrate and ultrathin substrate on the supporting glass. Black scale bar indicates 1 cm. (**B**) Transmittance of the glass substrate and free-standing parylene/SU-8 substrate. Surface images of glass and ultrathin substrate obtained using atomic force microscopy (AFM), (**C**) glass substrate, (**D**) glass/parylene/SU-8. The black scale bar of each image indicates 0.5 μm.

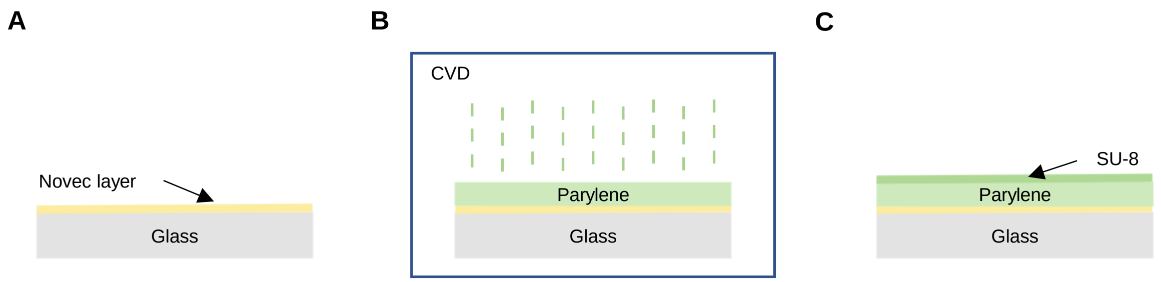

Fig. S8. Fabrication process of ultrathin substrate. (**A**) Spin coated Novec layer on the cleaned glass to change the surface energy for easy peeling. (**B**) Chemical vapor deposition (CVD) of the parylene substrate. (**C**) SU-8 spin-coating and UV curing. The total thickness of the ultrathin substrate is approximately 2 μm.

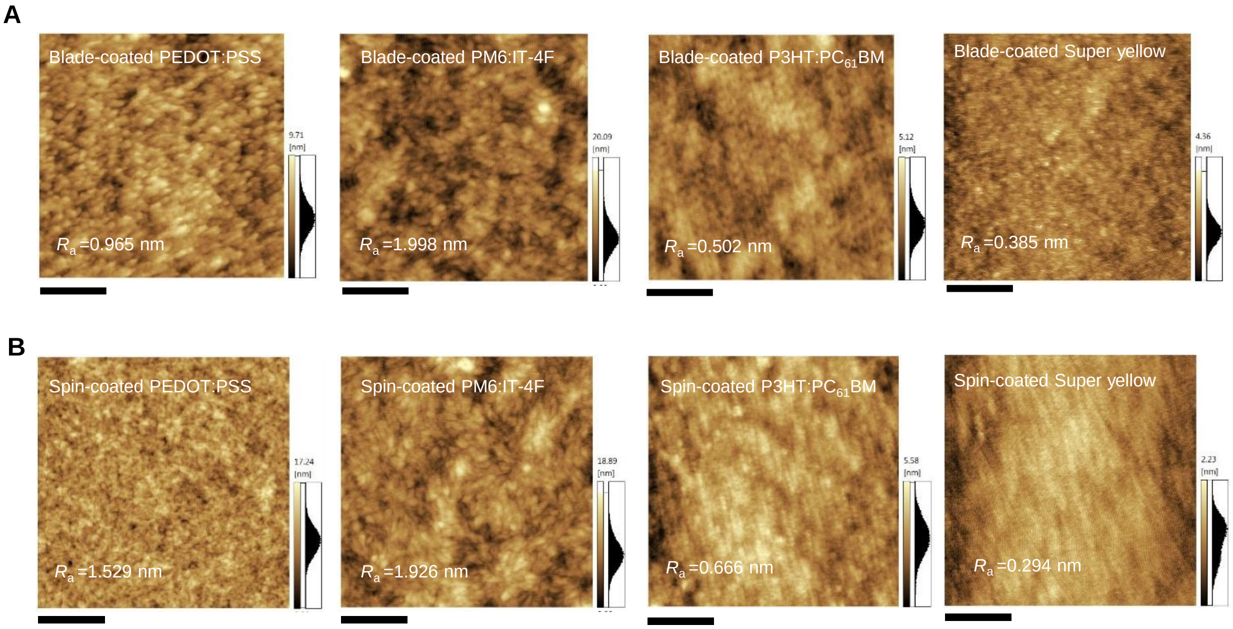

Fig. S9. Surface images of the polymer films obtained via AFM. (**A**) Surface images of blade-coated modified PEDOT:PSS, PM6:IT-4F, P3HT: $PC_{61}BM$, and Super Yellow layer on ultrathin substrate, respectively. The black scale bar of each image indicates 0.5 µm. (**B**) Surface images of spin-coated modified PEDOT:PSS, PM6:IT-4F, P3HT:$PC_{61}BM$, and Super Yellow layer on the glass substrate, respectively. The black scale bar of each image indicates 0.5 µm.

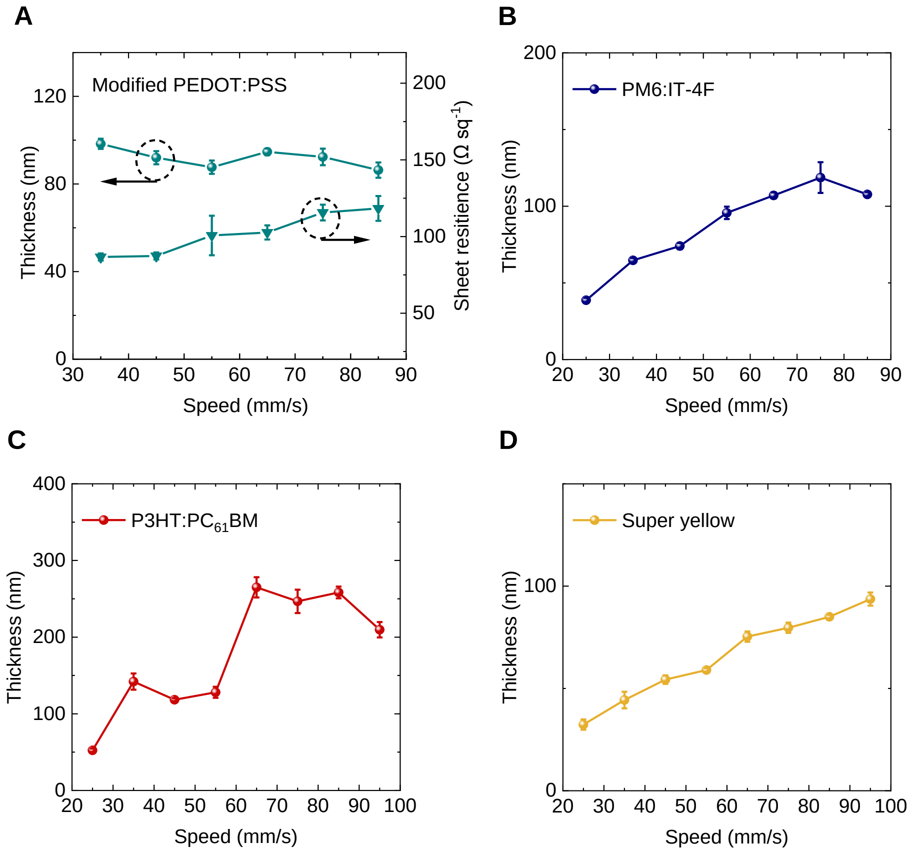

Fig. S10. Film thickness of the blade-coated organic functional layer. Organic functional films were prepared at different coating speeds. (**A**) Thickness and sheet resistance of the modified PEDOT:PSS film at different coating speeds. (**B**) Thickness of the active layer (PM6:IT-4F) at different coating speeds. (**C**) Thickness of the active layer (P3HT:$PC_{61}BM$) at different coating speeds. (**D**) Thickness of the active layer (Super Yellow) at different coating speeds.

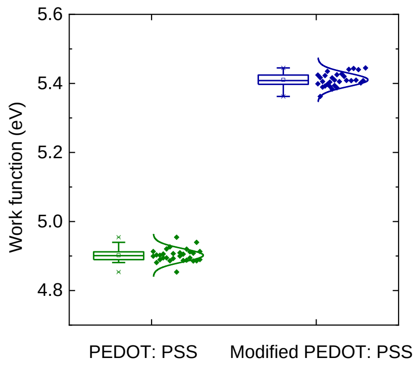

Fig. S11. Work function evaluation of PEDOT:PSS. Work function of pure PEDOT:PSS and modified PEDOT:PSS films. Thirty samples were fabricated for each type of PEDOT:PSS film.

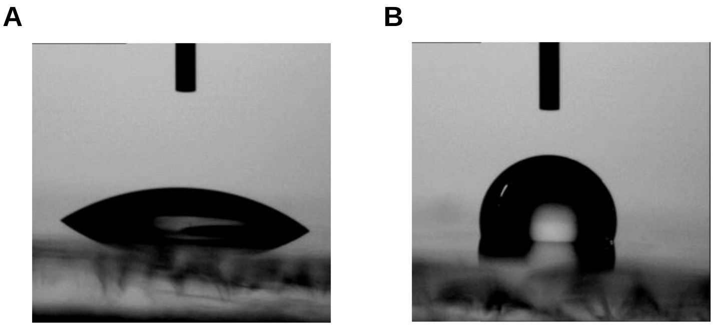

Fig. S12. Water contact angle of pure PEDOT:PSS and modified PEDOT:PSS films. Contact angle of water droplets on (**A**) pure PEDOT:PSS film is smaller than that of (**B**) modified PEDOT:PSS film.

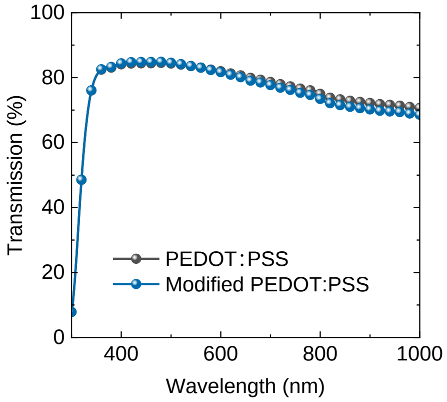

Fig. S13. Transmittance of PEDOT:PSS and modified PEDOT:PSS films. Films were deposited on the glass substrate and the thicknesses of the PEDOT:PSS and modified PEDOT:PSS films are approximately 100 nm.

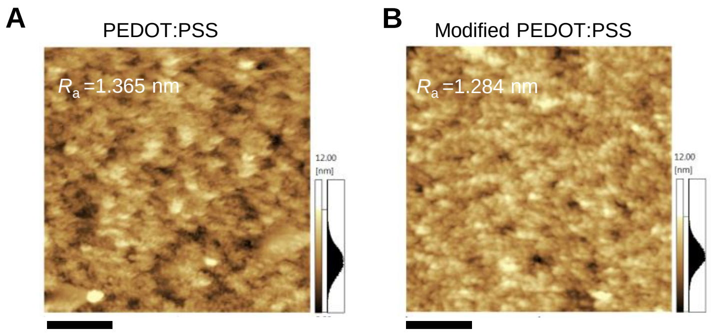

Fig. S14. Surface images of PEDOT:PSS films with AFM. Surface images of (**A**) PEDOT:PSS without additives on the glass substrate, and (**B**) modified PEDOT:PSS on the glass substrate, respectively. The black scale bar of each image indicates 0.5 μm.

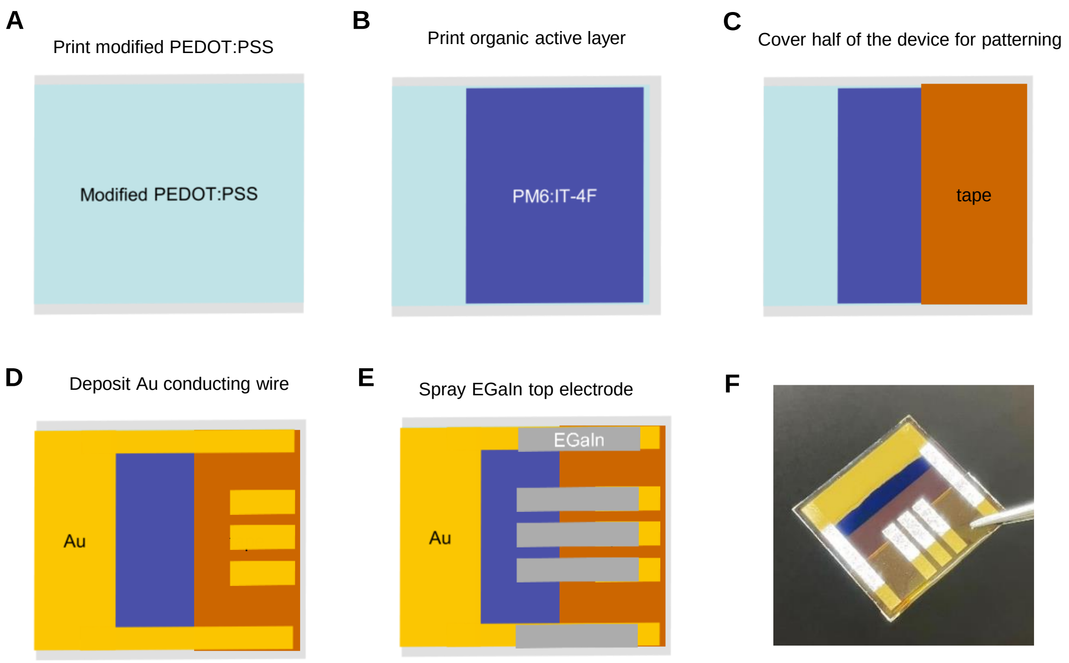

Fig. S15. Schematic of the fabrication process of all-solution processed devices. (**A**) Print modified PEDOT:PSS on the ultrathin substrate. (**B**) Print organic active layer: PM6:IT-4F, P3HT:PC_{61}BM, and Super Yellow. (**C**) Cover half of the device for patterning. (**D**) Deposit Au conducting wire. (**E**) EGaIn electrode sprayed on the top of the active layer with the shadow mask. (**F**) Photograph of the all-solution processed OPV.

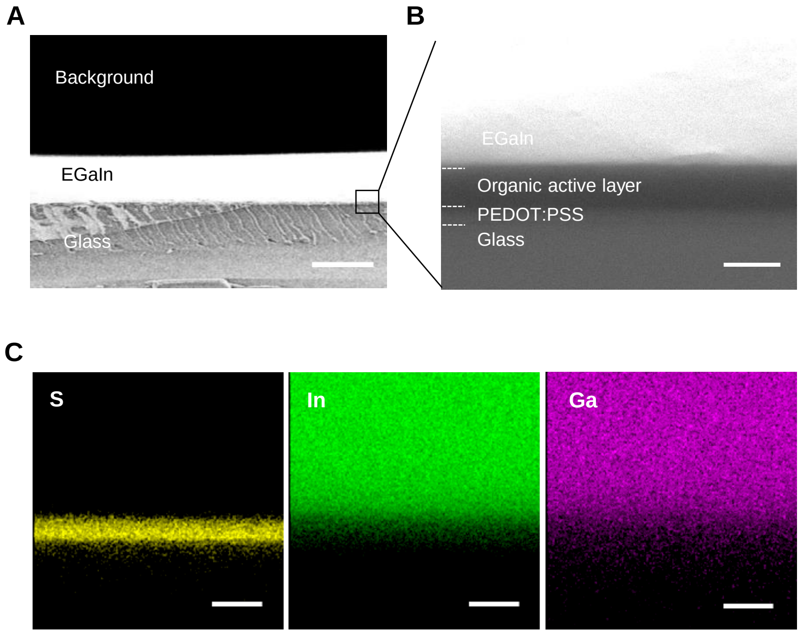

Fig. S16. Cross-section scanning electron microscopy (SEM) image. (**A**) Low-resolution cross-sectional SEM image of the all-solution processed organic optoelectronics device. We used the OPV device for the typical observation. The white scale bar indicates 10 µm. (**B**) High-resolution cross SEM image of the all-solution processed organic optoelectronics device. The white scale bar indicates 250 nm. (**C**) Cross-sectional SEM image with EDX maps showing the elemental chemical analysis of the all-solution processed organic optoelectronics device. The S element represents the active layer of PEDOT:PSS and organic active layer. The In and Ga elements represent the EGaIn layer. The white bar indicates 400 nm.

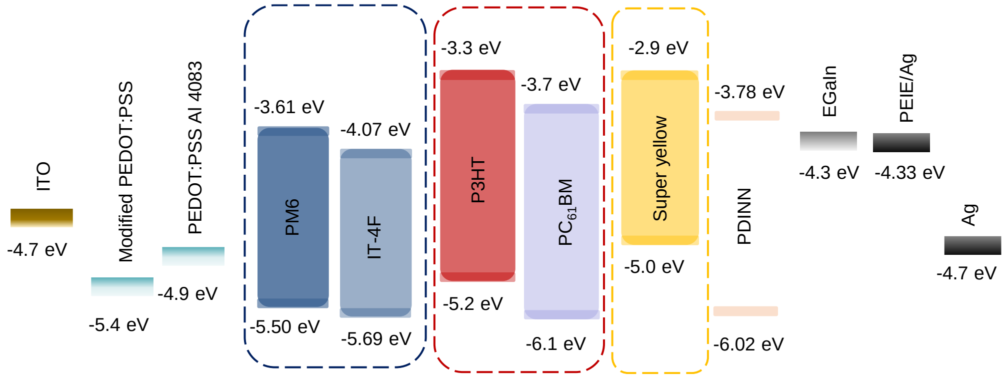

Fig. S17. Energy-level diagram of the organic optoelectronic devices. Energy levels of the organic optoelectronics devices based on vacuum-evaporation and all-solution methods.

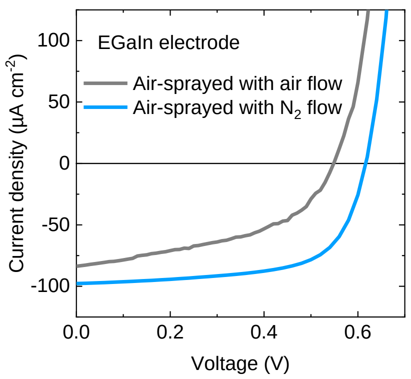

Fig. S18. Device performance of the all-solution processed OPVs with high-pressure air flow via spraying coating. *J*–*V* curves of the device evaluated under LED light (1000 lx).

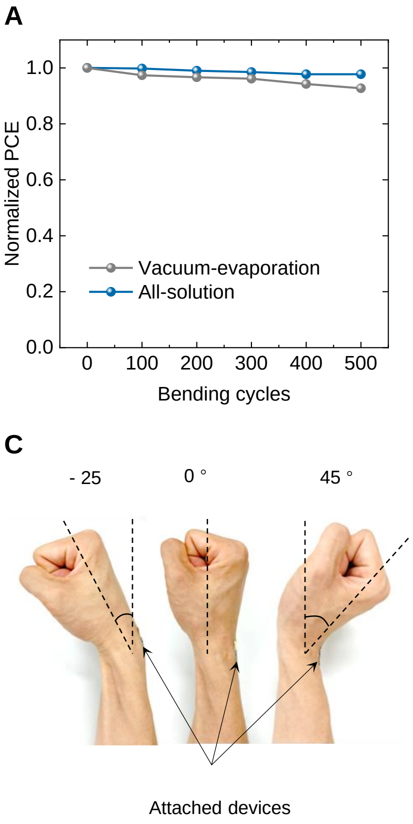

Fig. S19. Flexibility of the all-solution processed OPV device. (**A**) Evolution of the PCE of the all-solution processed OPVs and vacuum-evaporated reference after 500 bending cycles at a bending radius of 5 mm. (**B**) Mechanical flexibility of the sprayed liquid metal. Evolution of the resistance of the sprayed EGaIn film on the ultrathin substrate after 1000 bending cycles at a bending radius of 5 mm. (**C**) Photograph of the all-solution processed OPV attached to the wrist during bending. The degree of wrist bending is 70°. (**D**) Evolution of the PCE of the all-solution processed OPVs after the wrist bending cycles.

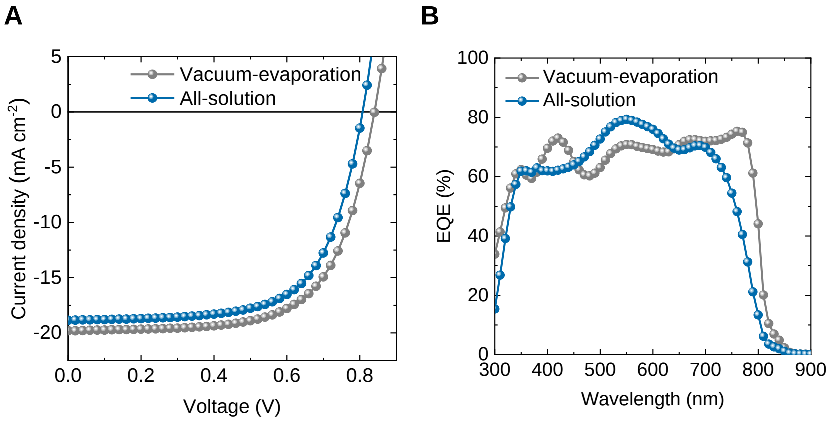

Fig. S20. Device performance of the all-solution processed single-junction OPVs under AM 1.5G (100 mW cm^{-2}). (**A**) *J*–*V* curves of the device. (**B**) EQE curves of the all-solution processed OPVs. The blue line indicates the all-solution processed OPVs with PEDOT:PSS/PM6:IT-4F/EGaIn structure. Vacuum-evaporated reference fabricated with the ITO/PEDOT:PSS/PM6:IT-4F/PDINN/Ag structure.

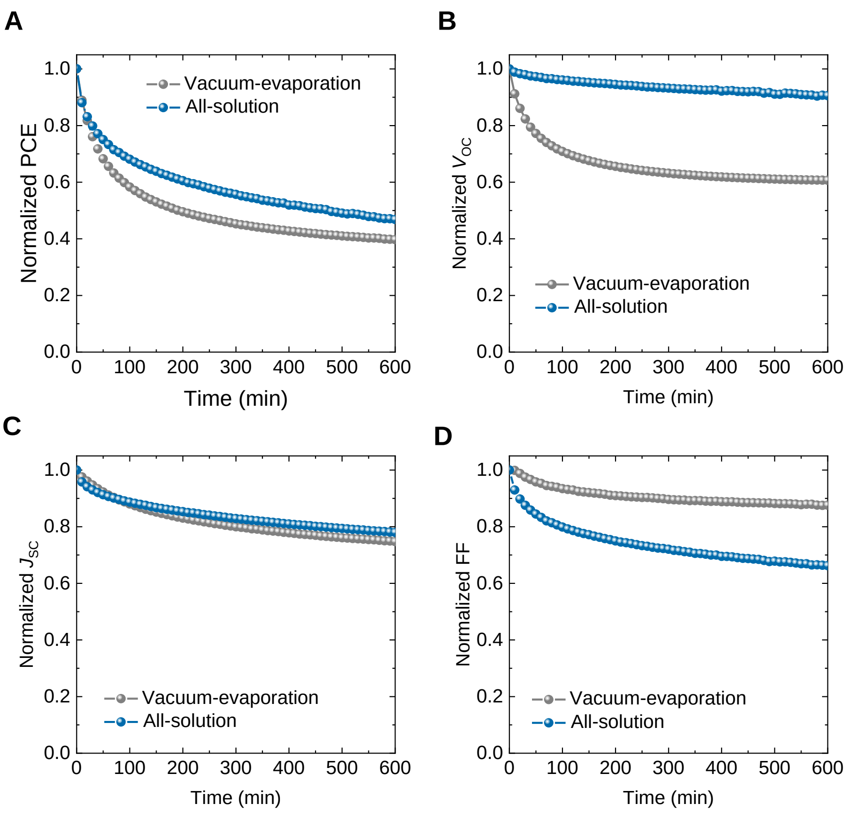

Fig. S21. Stability of the all-solution processed OPVs under AM 1.5G 1 Sun. (**A-D**) Normalized parameters of the 1-Sun MPP tracking of the device. The blue line represents the all-solution processed OPVs with the PEDOT:PSS/PM6:IT-4F/EGaIn structure. The black line represents a vacuum-evaporated reference with the ITO/PEDOT:PSS/PM6:IT-4F/PDINN/Ag structure. Devices were tested at a temperature of 25 °C in ambient air and without encapsulation.

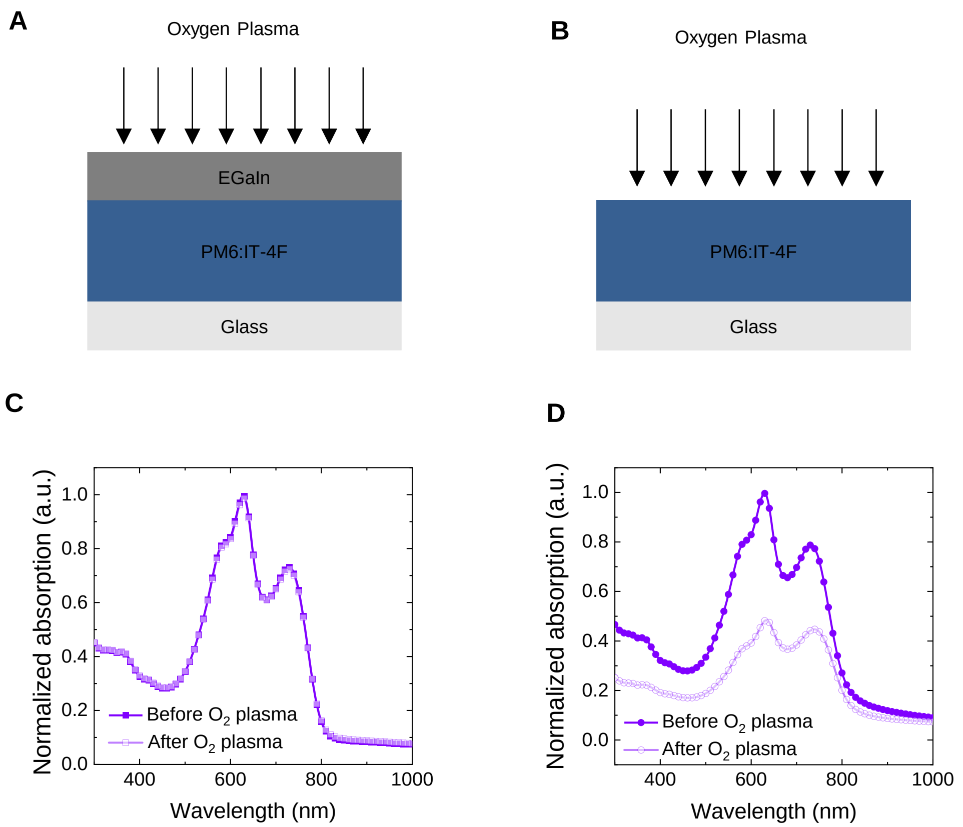

Fig. S22. Protection of the active layer by spraying EGaIn. (**A**) Schematic of oxygen plasma processing on an active layer deposited with EGaIn. (**B**) Schematic of oxygen plasma processing on an active layer without EGaIn. (**C**) Absorption changes in the active layer protected by EGaIn before and after plasma treatment. (**D**) Absorption changes in the active layer without EGaIn before and after plasma treatment. Oxygen power is 100 W, 1 min. Tape was used to remove EGaIn after plasma treatment.

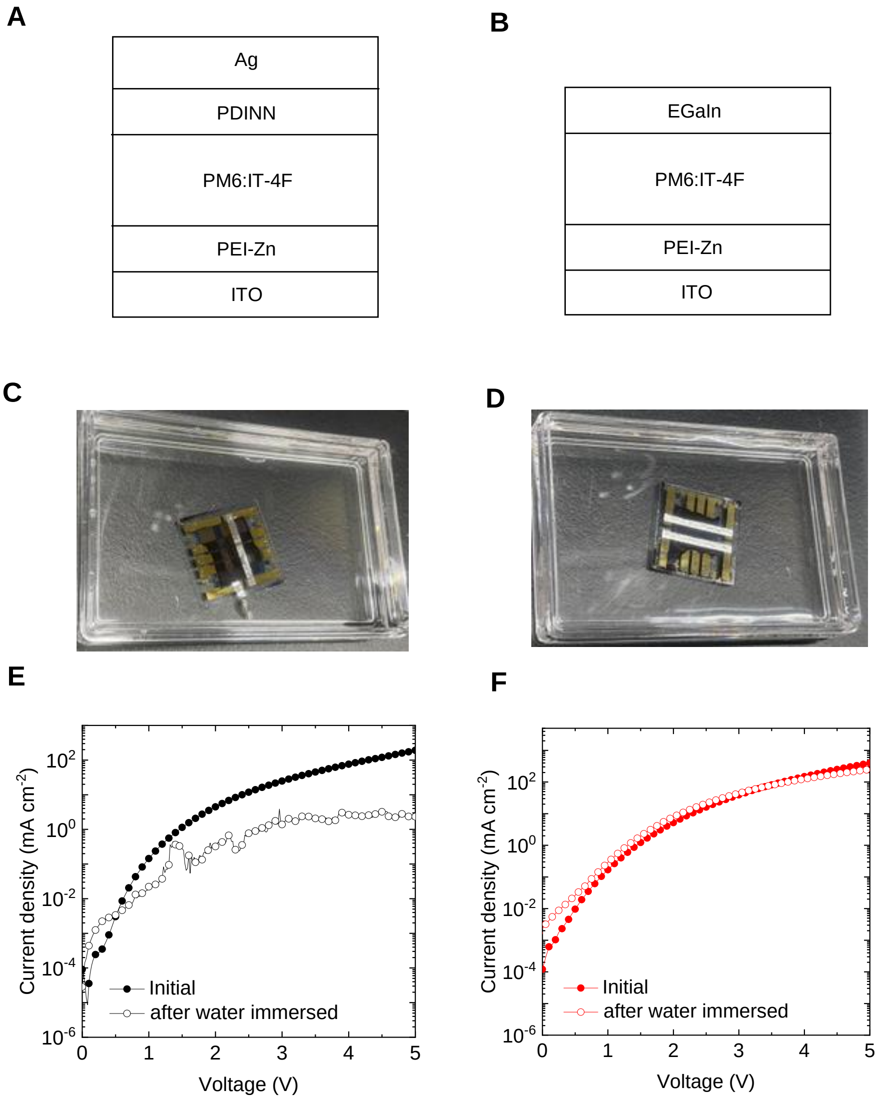

Fig. S23. Water stability of the sprayed EGaIn electrode. (**A**) Structure of the electron-only device with the PDINN/Ag electrode. (**B**) Structure of the electron-only device with the EGaIn electrode. (**C**) Photograph of the electron-only device with the PDINN/Ag electrode immersed in DI water. (**D**) Photograph of the electron-only device with the EGaIn electrode immersed in DI water. (**E**) *J–V* curves of the electron-only device with the PDINN/Ag electrode before and after immersing in DI water. (**F**) *J–V* curves of the electron-only device with the EGaIn electrode before and after immersing in DI water.

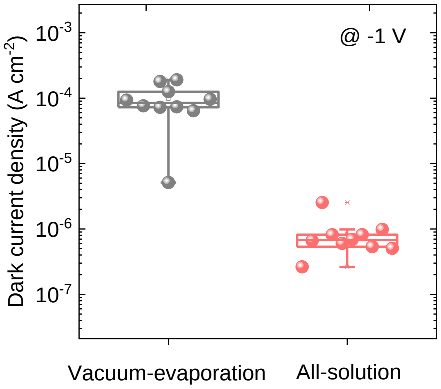

Fig. S24. Dark current density of all-solution processed OPDs. Histogram of the dark current density of all-solution processed and vacuum-evaporated OPDs.

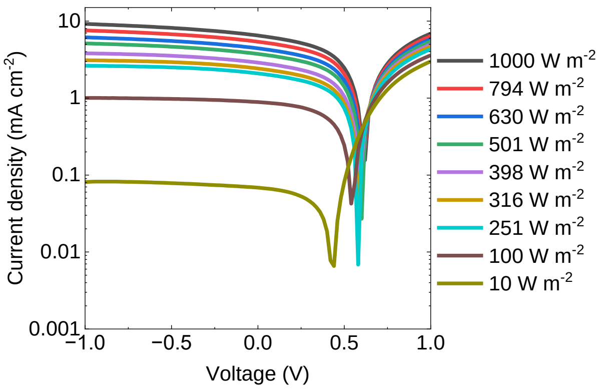

Fig. S25. Light intensity dependence of the all-solution processed OPDs. *J*–*V* curves of the all-solution processed OPDs under AM 1.5G 1-Sun illumination from a solar simulator. Light intensity of a solar simulator varies from 0.01 to 1-Sun with optical filters.

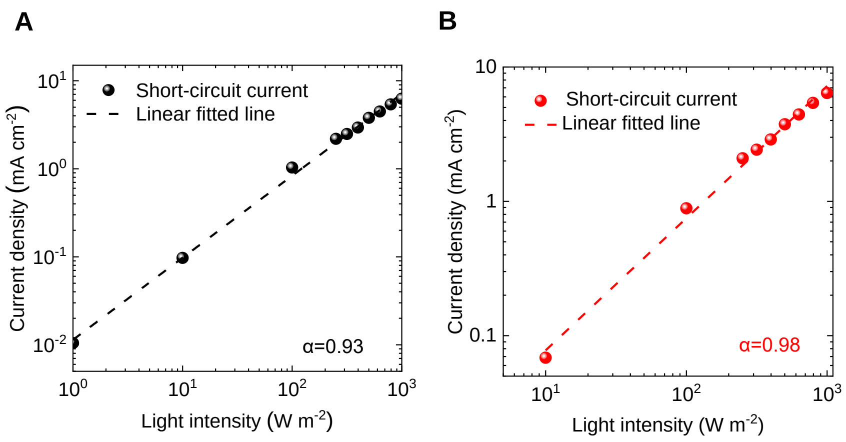

Fig. S26. Light intensity dependence of OPDs. (**A**) Current density of the vacuum-evaporated reference with different light intensities. (**B**) Current density of the all-solution processed devices with different light intensities. The light intensity of solar simulator varying from 0.01 to 1-Sun with optical filters. Dots represent vacuum-evaporated devices. The dashed line represents a linear fitted line of short-circuit current and light intensity. Additionally, α represents the light intensity exponent of OPDs with 1-Sun illumination.

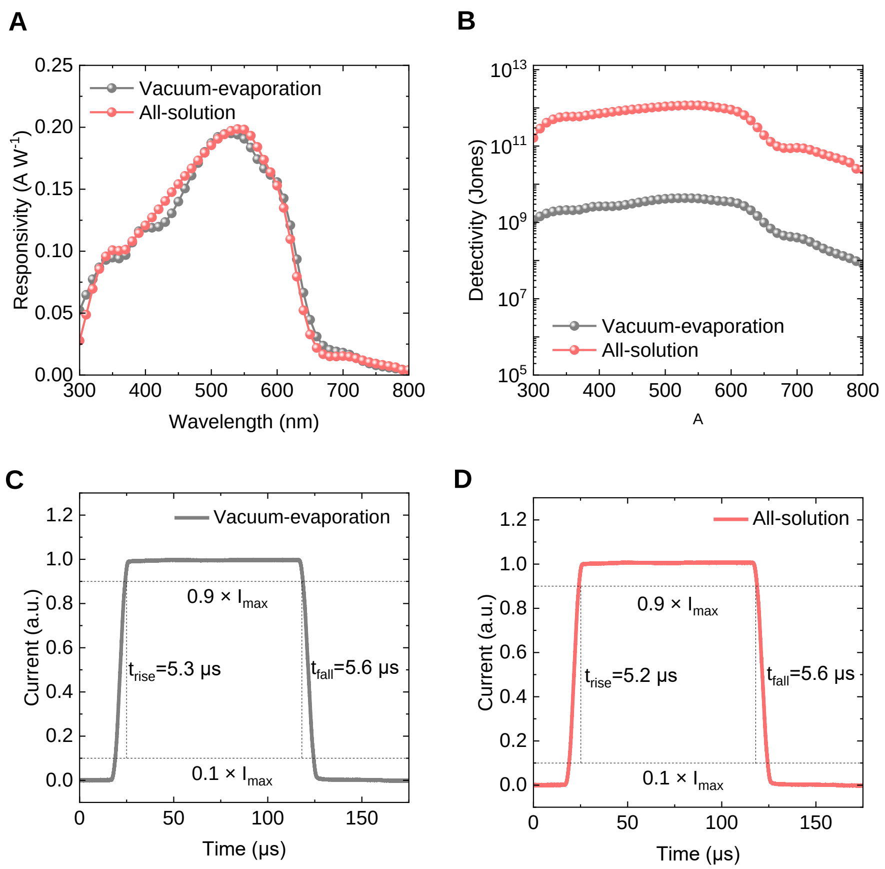

Fig. S27. Detailed characterization of the vacuum-evaporated and all-solution processed OPDs. (**A**) Responsivity of the vacuum-evaporated and all-solution processed OPDs. (**B**) Detectivity of the vacuum-evaporated and all-solution processed OPDs at 0 V. Time response of (**C**) vacuum-evaporated and (**D**) all-solution processed OPDs short-circuit current under light. A 550 nm laser was used as a light source and an optical chopper was operated at a frequency of 50 Hz.

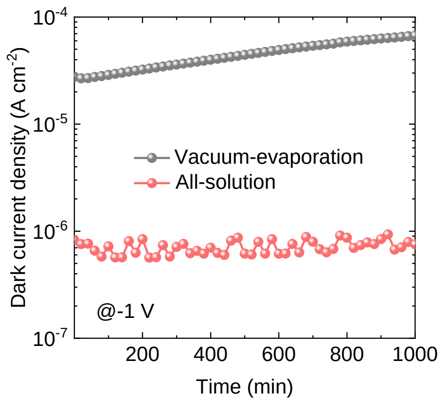

Fig. S28. Air stability of the all-solution processed OPDs. Dark current density changes in the OPDs stored under ambient air over time. The red line represents all-solution processed OPDs, and the black line represents the vacuum-evaporated reference. The devices were tested under dark ambient air without encapsulation.

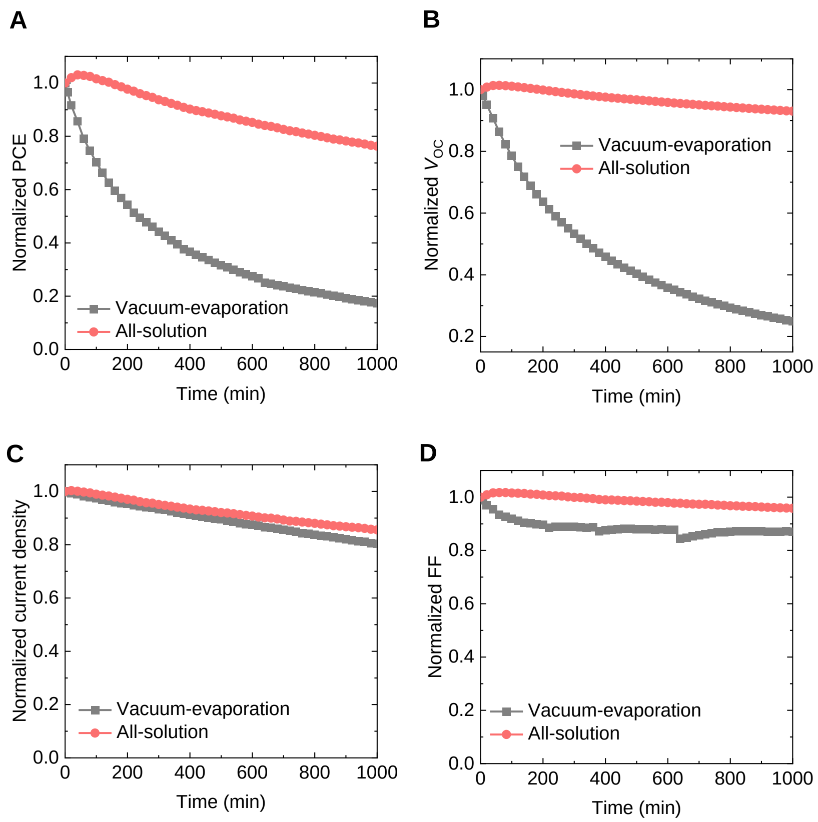

Fig. S29. Long-term storage stabilities of the OPDs under dark ambient air. (**A-D**) Normalized PCE, V_{OC}, current density, and FF under the illumination of 1-Sun light as a function of storage time. The red line represents the all-solution processed OPDs with the PEDOT:PSS/P3HT:$PC_{61}BM$/EGaIn structure. The black line represents a vacuum-evaporated reference with the ITO/PEDOT:PSS/P3HT:$PC_{61}BM$/PEIE/Ag structure. Devices were stored in dark ambient air and without encapsulation.

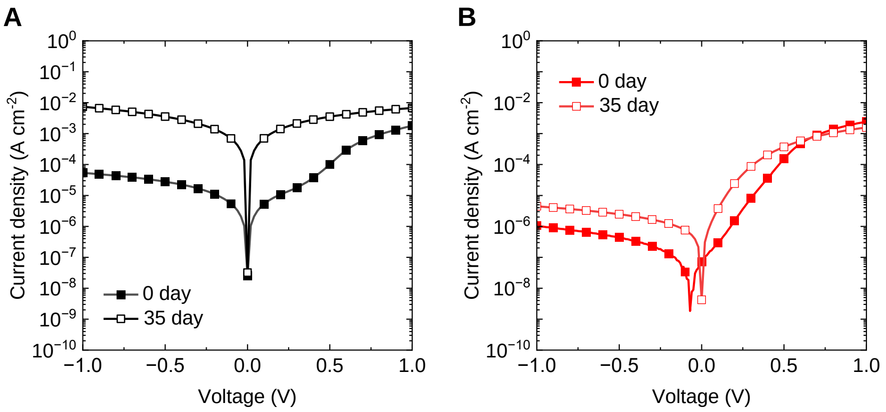

Fig. S30. Stability of the OPDs. (**A** and **B**) Change in the dark current density in (**A**) vacuum-evaporated and (**B**) all-solution processed OPDs. Devices were stored in dark ambient air without temperature and humidity control. Devices are encapsulated with 1-µm parylene.

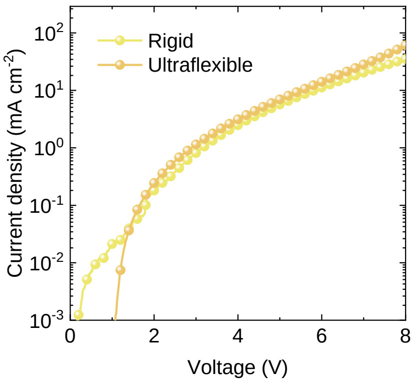

Fig. S31. *J-V* curves of the OLEDs. Current density–voltage curves of the all-solution processed OLED before and after peeling from the glass substrate.

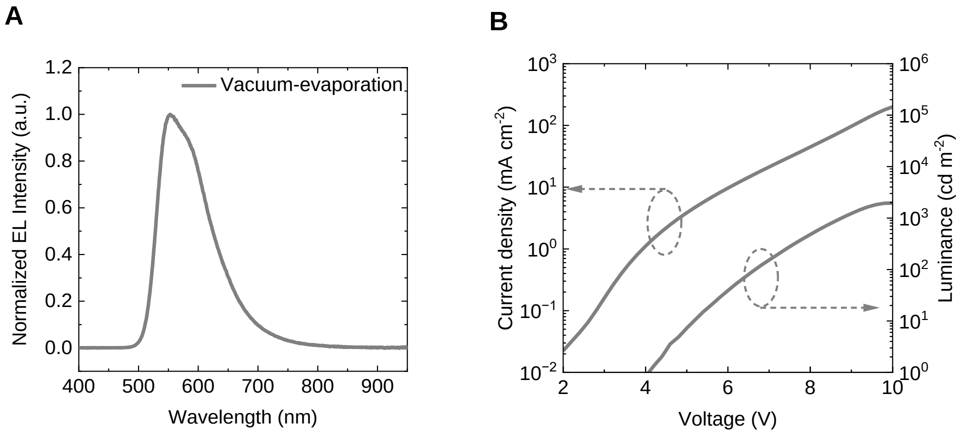

Fig. S32. Performance of the vacuum-evaporated OLEDs. (**A**) Normalized electroluminescence (EL) spectra of the vacuum-evaporated OLEDs with structure of ITO/PEDOT:PSS/SY/PEIE/Ag. (**B**) Current density-luminance-voltage curves of the vacuum-evaporated OLEDs.

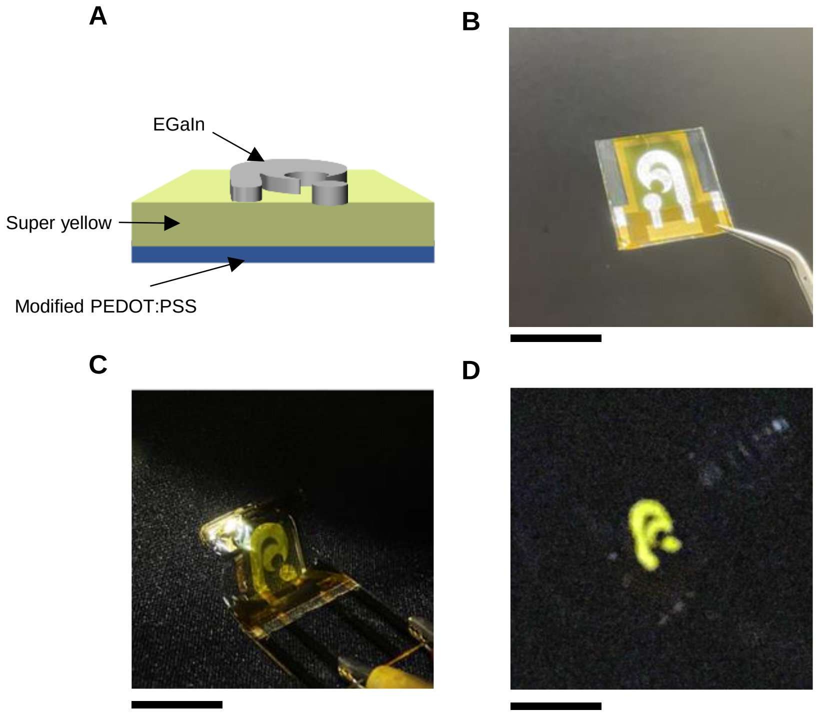

Fig. S33. Photograph demonstrating the all-solution processed OLEDs. (**A**) Schematic of the all-solution processed OLEDs. (**B**) Photograph of the all-solution processed OLEDs on a glass support. (**C**) Photograph of the free-standing all-solution processed OLEDs. (**D**) Photograph of the free-standing all-solution processed OLEDs applied 7 V. The scale bar indicates 2 cm.

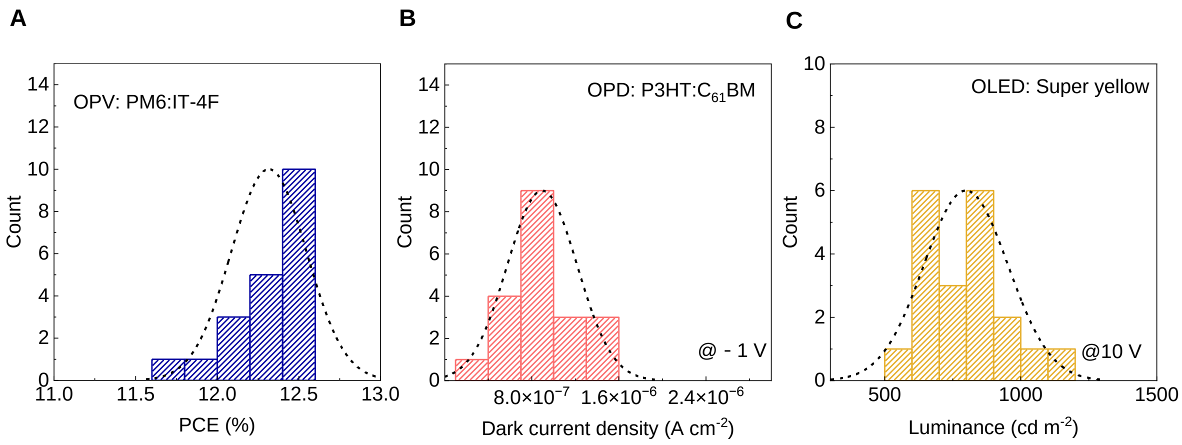

Fig. S34. Reproducibility of the all-solution processed organic optoelectronic devices. (**A**) Histogram of the PCE extracted from OPVs under LED light (1000 lx). (**B**) Histogram of the dark current density extracted from OPDs. (**C**) Histogram of the luminance extracted from OLEDs. Performance statistics were obtained from 10 batches of devices.

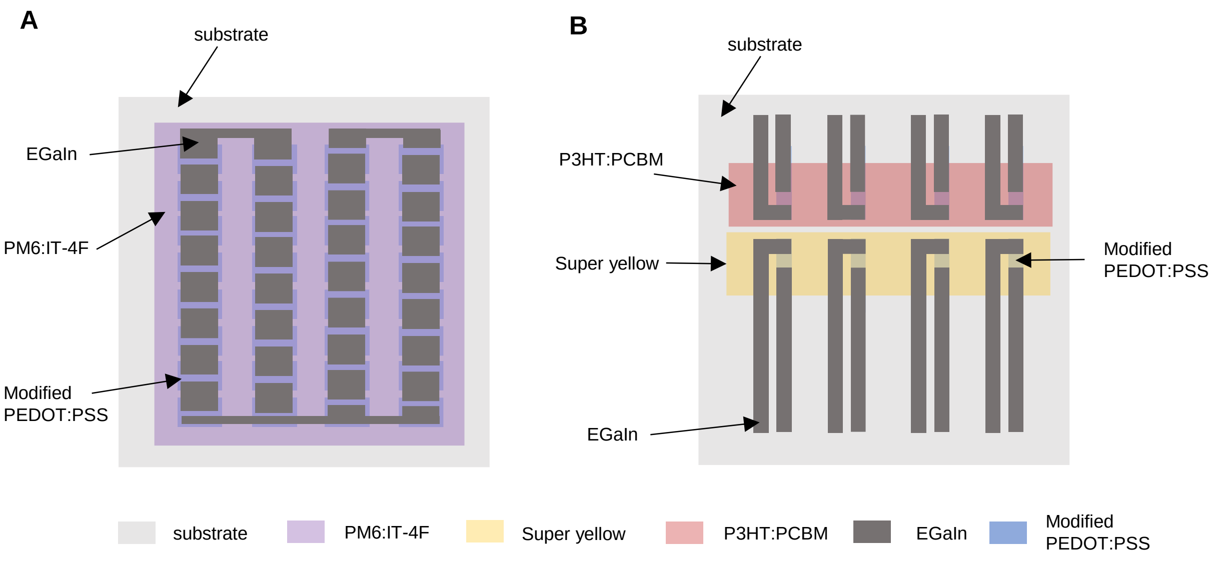

Fig. S35. Top-view schematic of the large-area all-solution processed organic optoelectronic devices. (**A**) Organic solar module. Size of the substrate is 5 × 5 cm. The module contains 32 single cells connected in parallel and series. The total area of the active layer is 3.2 cm^2. (**B**) Reflective-model PPG sensor. Each substrate contains four sensors. The sensor is combined by all-solution processed OLEDs and OPDs.

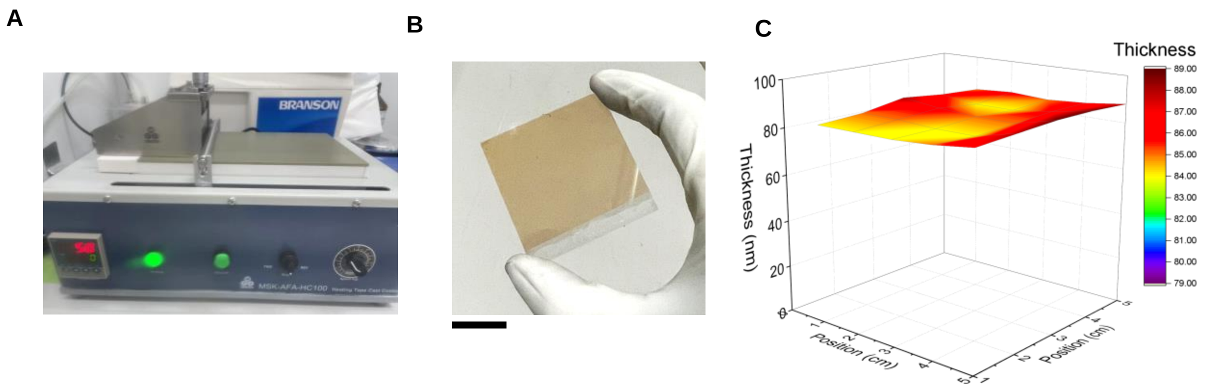

Fig. S36. Blade-coating technique for preparing large-area organic thin films. (**A**) Photograph of the blade-coating equipment. (**B**) Photograph of the blade-coated P3HT film. The scale bar indicates 2 cm. (**C**) Thickness of the blade-coated P3HT film at different positions on the glass substrate.

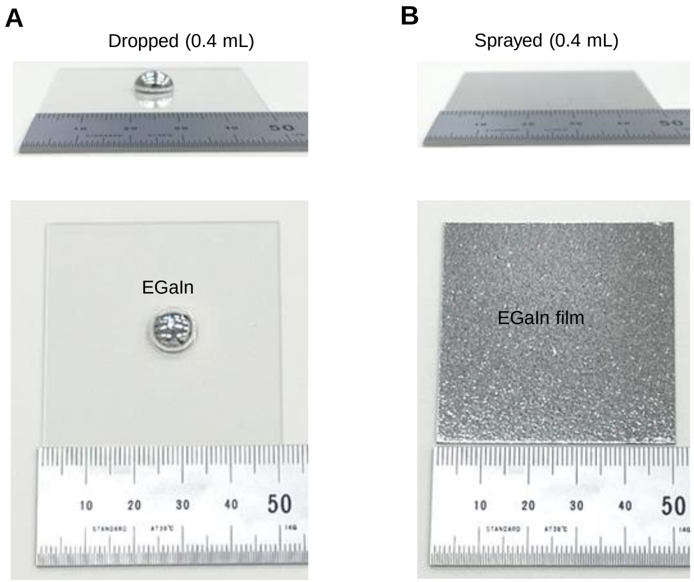

Fig. S37. Photographs of the large-area EGaIn film. (**A**) EGaIn dropped onto the surface of the glass. (**B**) EGaIn sprayed on the surface of the glass. Size of the glass substrate is 5 × 5 cm.

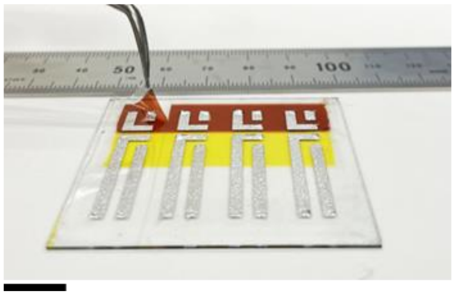

Fig. S38. Photograph of the all-solution processed PPG sensor. Peeling process of the ultraflexible all-solution processed sensors. One sensor is cut and peeled from the glass support. The black scale bar indicates 1 cm.

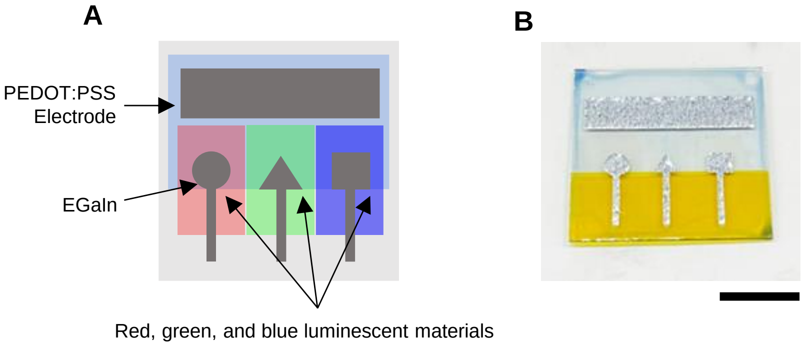

Fig. S39. Multicolor displays with different shapes. (**A**) Top-view schematic of the multicolor display device. (**B**) Photograph of the multicolor displays device. The scale bar indicates 1 cm.

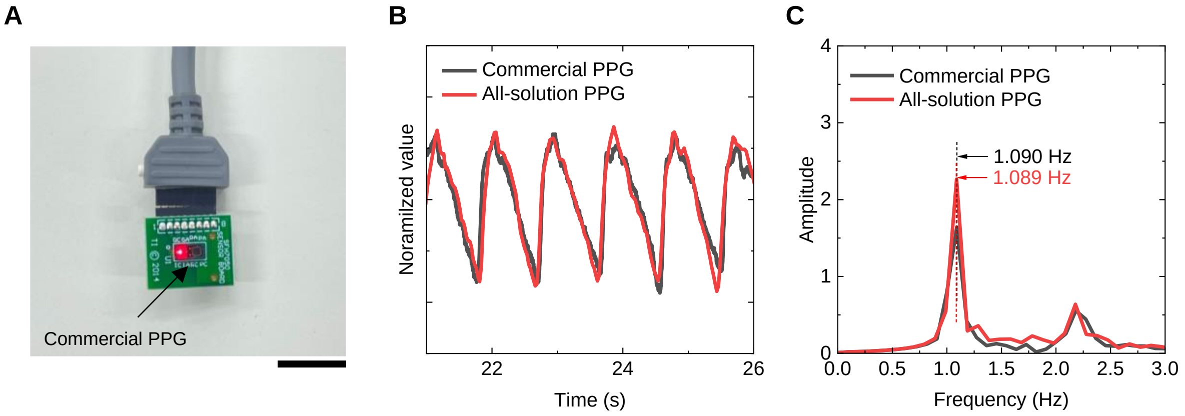

Fig. S40. Comparison with commercial PPG signal. (**A**) Photograph of a commercial PPG device (Texas Instruments Inc., AFE44403). (**B**) Normalized values of the signal measured from commercial PPG devices and all-solution processed PPG devices. (**C**) Blood pulse frequency is 66 bpm from the measurement results of both commercial and all-solution processed PPG devices. The detection was performed simultaneously to ensure the accuracy of the test.

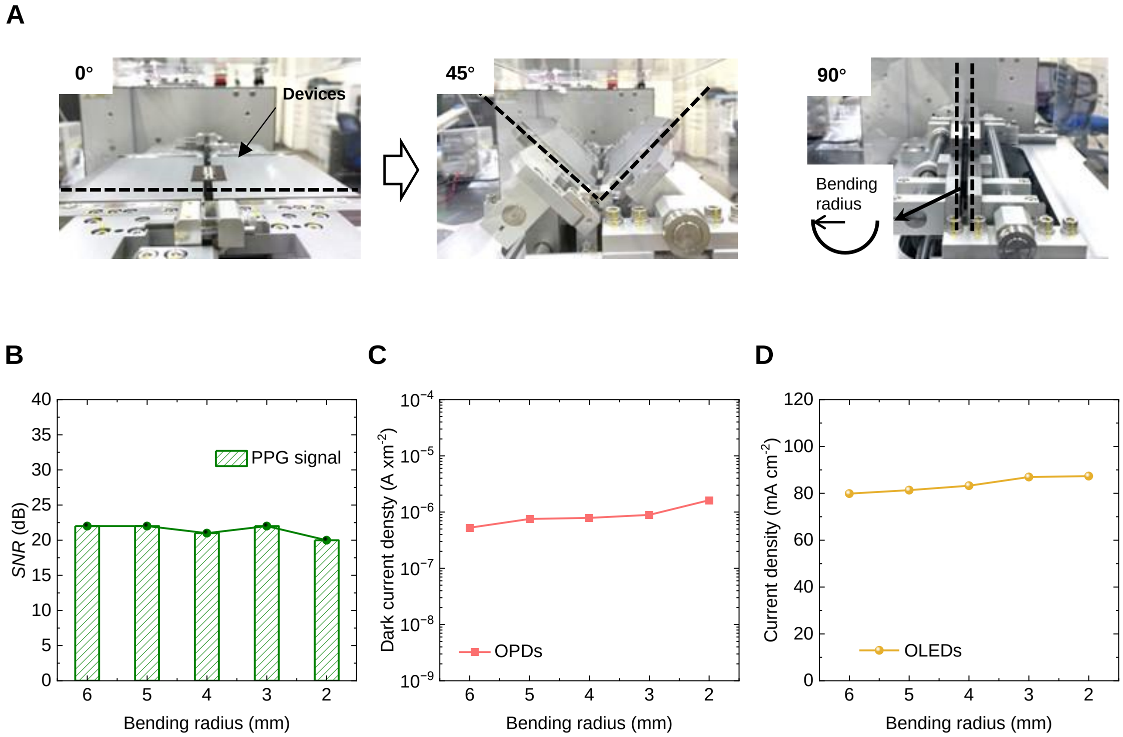

Fig. S41. Flexibility of the all-solution PPG devices based on OPDs and OLEDs. (**A**) Photographs of the bending test settings. Each bending radius was tested for 100 cycles. (**B**) Signal-to-noise ratio (SNR) of the PPG sensor after the bending cycles test. (**C**) Dark current density changes of the OPDs after bending cycles test. (**D**) Current density changes of the OLEDs operated at 10 V after bending cycles.

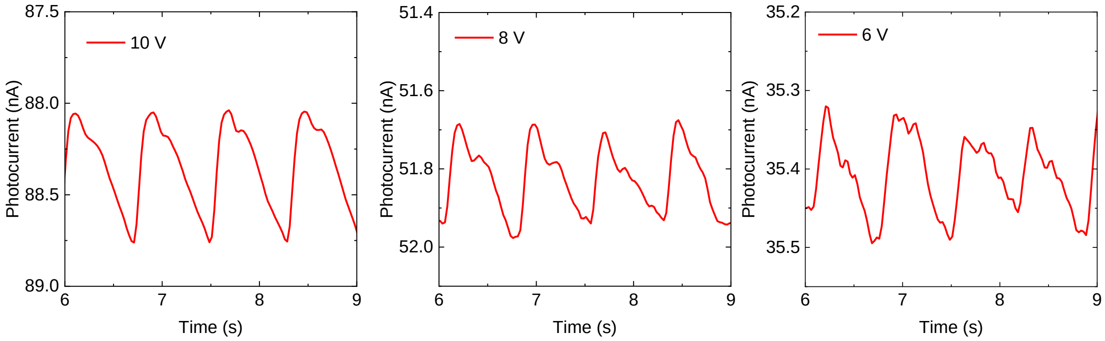

Fig. S42. All-solution processed PPG signals. All-solution processed electronic system at a different driving voltage of OLEDs. Driving voltage of OLEDs in the PPG is changed from 6 V to 10 V.

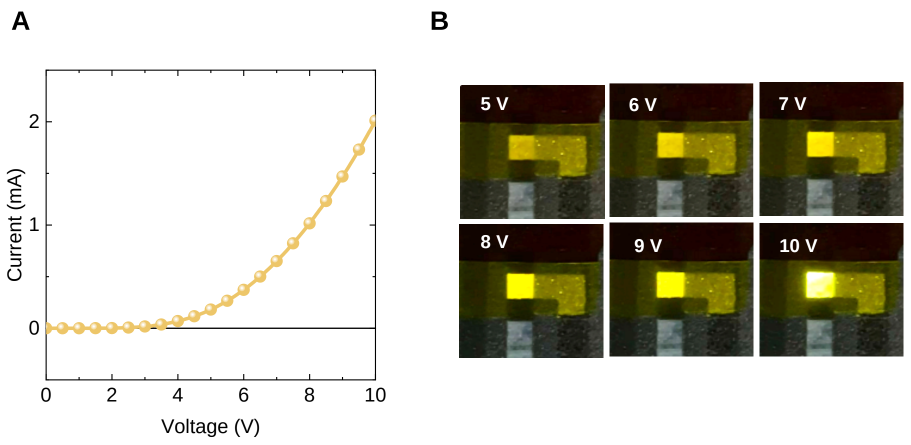

Fig. S43. All-solution processed OLEDs in the PPG sensor. (**A**) *I*–*V* curves of the all-solution processed OLEDs in the PPG sensor. (**B**) Photographs of the all-solution processed OLEDs in the PPG sensor driving at a different voltage.

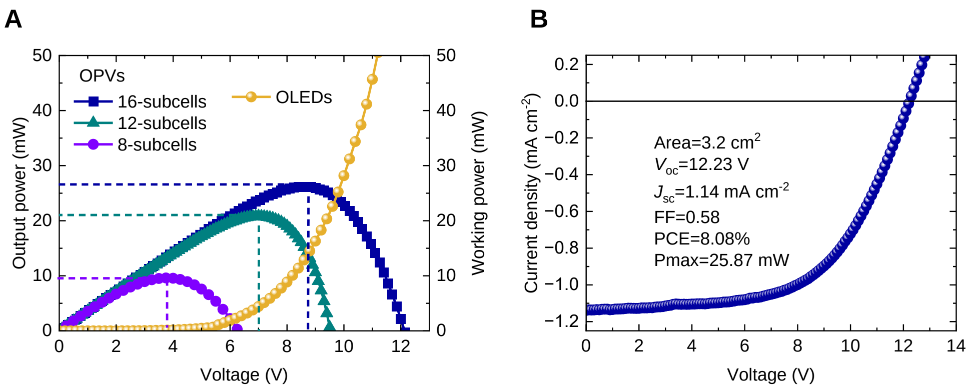

Fig. S44. All-solution processed solar module. (**A**) Output power characteristics of 8, 12, and 16 series connected OPV modules. (**B**) *J*-*V* curve of the all-solution processed solar module.

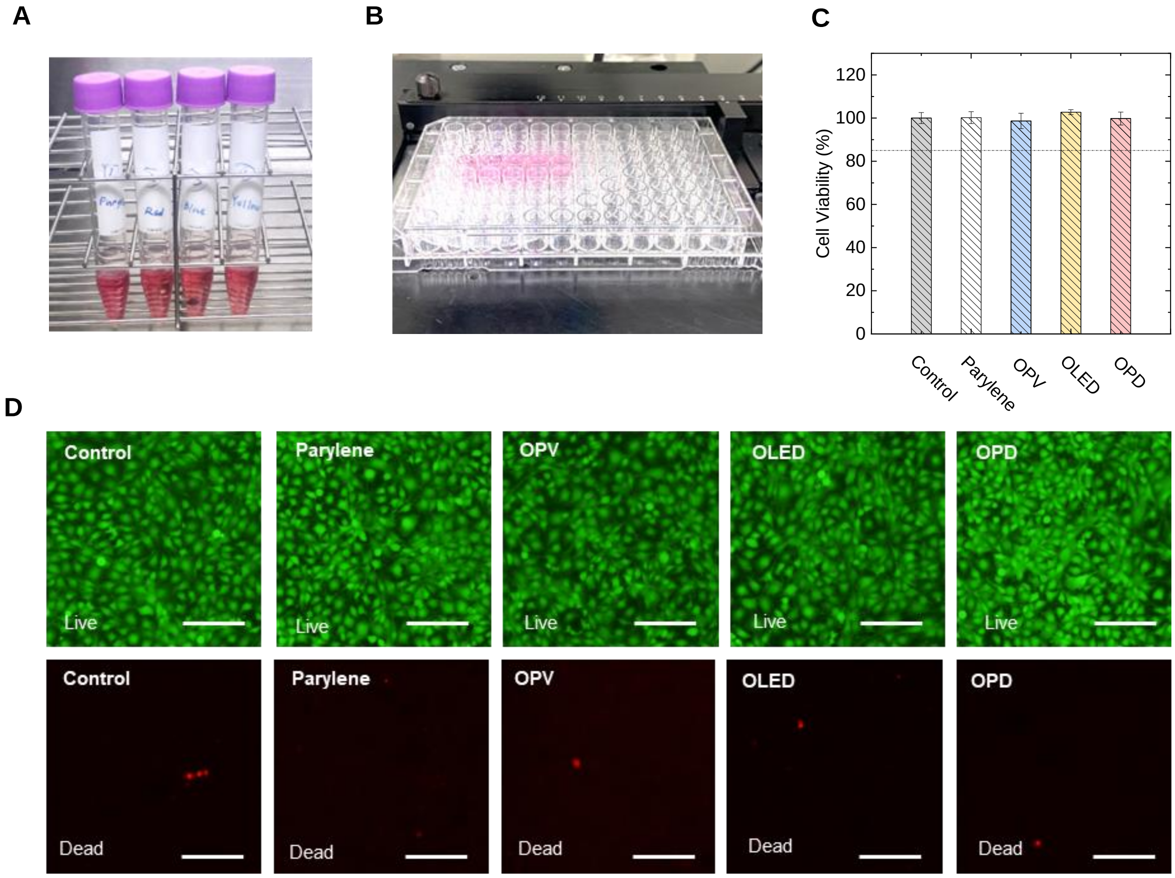

Fig. S45. Biocompatibility of the device. (**A**) All-solution processed organic photoelectric device and parylene layer are soaked in DMEM for 24 h. (**B**) NIH3T3 cells culturing in 96-well plate. Fresh DMEM was used as a control. (**C**) Cell viability after 48 h co-culture in the extracts. (**D**) Fluorescence microscopy images of NIH3T3 cells after 48 h culture in the extracts. Green: live cells. Red: dead cells. The white scale bar indicates 100 μm.

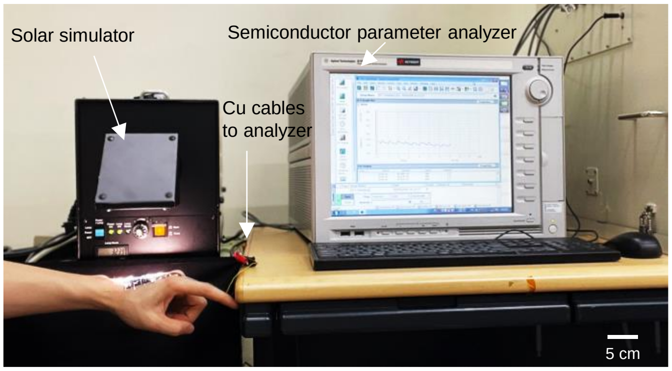

Fig. S46. The Steup for all-solution processed PPG signals. The semiconductor parameter analyzer (B1500A, Keysight) is used to process and display the obtained heart pulse wave signal in real time. The solar simulator is used to enable the organic photovoltaic to generate operating power. The self-powered health monitoring electronics system is attached to the skin surface and connected to the semiconductor device via Cu cables.

Table S1. Summary of the air-processed EGaIn in organic photovoltaics.

Device structure	Extra interface	Deposition environment	Deposition technique	Available area	Substrate	Efficiency (%)	V_{OC} (V)	Current density (mA cm^{-2})	FF	Ref.
PEDOT:PSS/PM6:IT-4F/EGaIn	No	Ambient air	Spray (N_2 flow)	3.2 cm^2<br>0.04 cm^2	Ultrathin	8.08<br>9.96	12.23<br>0.81	1.14<br>18.86	0.58<br>0.65	Our
PH1000/Al 4083/active layer/PNDIT-F3N-Br/EGaIn	PNDIT-F3N-Br	Ambient air	Spray (Air flow)	0.164 cm^2	TPU	11.2	0.82	21.1	0.65	(*65*)
PH1000/Al 4083/active layer/PNDIT-F3N-Br/EGaIn	PNDIT-F3N-Br	Ambient air	Spray (Air flow)	0.09 cm^2	TPU	11.16	0.81	21.12	0.66	(*66*)
ITO/PEDOT:PSS/AnE-PVab:PCBM/ZnO/EGaIn	ZnO	Ambient air	Brush painting	-	Glass	1.93	0.83	4.70	0.50	(*67*)
ITO/PEDOT:PSS/PTB7:PCBM/PEO/EGaIn	PEO	Ambient air	Blade-coating	0.09	Glass	3.1	0.70	14.1	0.30	(*68*)
ITO/TiO_2/P3HT:PCBM/V_2O_5/EGaIn	V_2O_5	Ambient air	Brush-painting	0.1	Glass	0.38	0.58	2.50	0.27	(*69*)

Table S2. Photovoltaic parameters of evaporated and all-solution OPVs under LED light.

Light source		V_{oc} (V)	J_{sc} (μA cm^{-2})	FF	P_{max} (μW cm^{-2})	P_{in} (μW cm^{-2})	PCE (%)
1000 lx	All-solution	0.62	97.72	0.65	39.38	311.94	12.78 (12.71 ± 0.06)[a]
	Vacuum-evaporation	0.62	100.74	0.66	41.22	311.94	13.38 (13.28 ± 0.1)[a]
500 lx	All-solution	0.58	48.52	0.62	17.44	155.24	11.32 (11.02 ± 0.2)[a]
	Vacuum-evaporation	0.58	51.53	0.64	19.12	155.24	12.41 (12.14 ± 0.24)[a]
200 lx	All-solution	0.57	20.22	0.60	6.91	59.82	11.54 (11.03 ± 0.47)[a]
	Vacuum-evaporation	0.57	22.15	0.60	7.57	59.82	12.67 (12.35 ± 0.29)[a]

[a] Averaged over five devices.

Table S3. Performance parameters of evaporated and all-solution OPD devices.

Devices	V_{oc} (V)	J_{sc} (mA cm^{-2})	FF	PCE (%)	Dark current density (A cm^{-2})	Responsivity (A W^{-1})	Detectivity (Jones)
Vacuum-evaporation	0.61	6.40	0.40	1.57	4.69×10^{-6} @ -1 V	0.190 @ 550 nm	4.20×10^{9} @ 550 nm
All-solution	0.60	6.34	0.39	1.49	2.01×10^{-7} @ -1 V	0.198 @ 550 nm	1.16×10^{12} @ 550 nm

Table S4. Performance parameters of evaporated and all-solution OLED devices.

Devices	Peak wavelength (nm)	CIE	Turn-on voltage (V)	Luminance (cd m^{-2})
Vacuum-evaporation	555	(0.473, 0.519)	4.0	1956 @ 10 V
All-solution	554	(0.478, 0.513)	3.8	1166 @ 10 V

Table S5. Summary of the reported PPG sensor based on organic optoelectronics.

Device structure	Fabrication process	Substrate thickness	SNR (dB)	Power source	Ref.
**OPD** and **OLED**: PEDOT:PSS/Organic layer/EGaIn	All-solution processing	2 μm	23	OPV-25.87 mW	*our*
**OLED**: ITO/Hole transport layer/organic layer/NaF/Al<br>**OPD**: ITO/P3HT:PCBM/MoOx/Au	Vacuum-evaporation and solution-processing	3 μm	-	12 mA/cm^2	*(15)*
**OLED**: ITO/PEDOT:PSS/SY/Ca/Al<br>**OPD**: ITO/PEDOT:PSS/PTB7:PCBM/Al	Vacuum-evaporation and solution-processing	~100 μm	-	-	*(29)*
**OLED**: IZO/TAPC/NPB:Ir(MQD)$_2$acac:B3PYMPM/Li/Al<br>**OPD**: IZO/HAT-CN/C_{70}:TAPC/BmPyPB/Li/Al	Vacuum-evaporation	50 μm	-	5V, 25 μA	*(30)*
**OLED**: ITO/PEDOT:PSS/F8BT:TFB/LiF/Al<br>**OPD**: Conductive PEDOT:PSS/PEDOT:PSS/PTB7:PC_{71}BM/Al	Vacuum-evaporation and solution-processing	~125 μm	-	9 V-battery	*(31)*
**OLED**: ITO/PEDOT:PSS/OLED AM/Ca/Al<br>**OPD**: PEDOT:PSS/OPD AM/Al	Vacuum-evaporation and solution-processing	125 μm	-	10 mA cm^{-2} with a 9-V battery	*(34)*
**OPD** and **OLED**: IZO/PDL/organic layer/Al	Vacuum-evaporation and solution-processing	5 μm	20	-	*(64)*
**OPD** and **OLED**: IZO/PEDOT:PSS/organic layer/LiF/Al	Vacuum-evaporation and solution-processing	120 μm	-	-	*(70)*
**OLED**: ITO/MoO_3/TAPC/TCTA:Ir(mppy)$_3$/TPBi/LiF/Al<br>**OPD**: ITO/MoO_3/DMQA/DCV3T/TPBi/LiF/Al	Vacuum-evaporation	1000 μm	-	OLED-60 μA	*(71)*
**OLED**: ITO/PEDOT:PSS/NPB/DPVBi:BCzVBi/BPhen/LiF/Al<br>**OPD**: ITO/PEDOT:PSS/CuPc/C60/BPhen/LiF/Al	Vacuum-evaporation and solution-processing	200 μm	-	15 V, 1.2 mA	*(72)*
**OLED:** IZO/NDP9:BCFA/BCFA/NET-164:Liq/Al<br>**OPD**: IZO/BCFA/SubNc/C_{60}/BPen/Al	Vacuum-evaporation	15 μm	21	3.8 V battery	*(73)*

Movie S1. Droplet of EGaIn falling in N_2 atmosphere.

A movie of EGaIn falling from a height of 20 cm onto a glass substrate with a deposited layer of PM6:IT-4F. Once liquid metal comes into contact with an organic semiconductor thin film in a nitrogen atmosphere, it experiences a squeezing rebound and eventually stabilizes, standing on the surface of the organic semiconductor.

Movie S2. Droplet of EGaIn falling in ambient air.

A movie of EGaIn falling from a height of 20 cm onto a glass substrate with a deposited layer of PM6:IT-4F. Once liquid metal comes into contact with an organic semiconductor thin film in ambient air, it immediately spreads and adheres to the surface of the organic semiconductor.

REFERENCES AND NOTES

1. Y. S. Rim, S.-H. Bae, H. Chen, N. De Marco, Y. Yang, Recent progress in materials and devices toward printable and flexible sensors. *Adv. Mater.* **28**, 4415–4440 (2016).

2. B. Wang, A. Facchetti, Mechanically flexible conductors for stretchable and wearable e-skin and e-textile devices. *Adv. Mater.* **31**, e1901408 (2019).

3. Y. Yang, W. Gao, Wearable and flexible electronics for continuous molecular monitoring. *Chem. Soc. Rev.* **48**, 1465–1491 (2019).

4. Y. Song, D. Mukasa, H. Zhang, W. Gao, Self-powered wearable biosensors. *Acc. Mater. Res.* **2**, 184–197 (2021).

5. H. Bronstein, C. B. Nielsen, B. C. Schroeder, I. McCulloch, The role of chemical design in the performance of organic semiconductors. *Nat. Rev. Chem.* **4**, 66–77 (2020).

6. C. Yan, S. Barlow, Z. Wang, H. Yan, A. K. Y. Jen, S. R. Marder, X. Zhan, Non-fullerene acceptors for organic solar cells. *Nat. Rev. Mater.* **3**, 18003 (2018).

7. Y. Liu, C. Li, Z. Ren, S. Yan, M. R. Bryce, All-organic thermally activated delayed fluorescence materials for organic light-emitting diodes. *Nat. Rev. Mater.* **3**, 18020 (2018).

8. F. P. García de Arquer, A. Armin, P. Meredith, E. H. Sargent, Solution-processed semiconductors for next-generation photodetectors. *Nat. Rev. Mater.* **2**, 16100 (2017).

9. G. Li, W.-H. Chang, Y. Yang, Low-bandgap conjugated polymers enabling solution-processable tandem solar cells. *Nat. Rev. Mater.* **2**, 17043 (2017).

10. B. Russ, A. Glaudell, J. J. Urban, M. L. Chabinyc, R. A. Segalman, Organic thermoelectric materials for energy harvesting and temperature control. *Nat. Rev. Mater.* **1**, 16050 (2016).

11. H. Jinno, K. Fukuda, X. Xu, S. Park, Y. Suzuki, M. Koizumi, T. Yokota, I. Osaka, K. Takimiya, T. Someya, Stretchable and waterproof elastomer-coated organic photovoltaics for washable electronic textile applications. *Nat. Energy* **2**, 780–785 (2017).

12. M. Kaltenbrunner, M. S. White, E. D. Głowacki, T. Sekitani, T. Someya, N. S. Sariciftci, S. Bauer, Ultrathin and lightweight organic solar cells with high flexibility. *Nat. Commun.* **3**, 770 (2012).

13. S. Park, S. W. Heo, W. Lee, D. Inoue, Z. Jiang, K. Yu, H. Jinno, D. Hashizume, M. Sekino, T. Yokota, K. Fukuda, K. Tajima, T. Someya, Self-powered ultra-flexible electronics via nano-grating-patterned organic photovoltaics. *Nature* **561**, 516–521 (2018).

14. M. Vosgueritchian, J. B. H. Tok, Z. Bao, Light-emitting electronic skin. *Nat. Photon.* **7**, 769–771 (2013).

15. T. Yokota, P. Zalar, M. Kaltenbrunner, H. Jinno, N. Matsuhisa, H. Kitanosako, Y. Tachibana, W. Yukita, M. Koizumi, T. Someya, Ultraflexible organic photonic skin. *Sci. Adv.* **2**, e1501856 (2016).

16. T. Yan, Z. Li, F. Cao, J. Chen, L. Wu, X. Fang, An all-organic self-powered photodetector with ultraflexible dual-polarity output for biosignal detection. *Adv. Mater.* **34**, e2201303 (2022).

17. Z. Liu, K. Parvez, R. Li, R. Dong, X. Feng, K. Müllen, Transparent conductive electrodes from graphene/PEDOT:PSS hybrid inks for ultrathin organic photodetectors. *Adv. Mater.* **27**, 669–675 (2015).

18. C. J. Brabec, J. R. Durrant, Solution-processed organic solar cells. *MRS Bull.* **33**, 670–675 (2008).

19. J. E. Carlé, M. Helgesen, O. Hagemann, M. Hösel, I. M. Heckler, E. Bundgaard, S. A. Gevorgyan, R. R. Søndergaard, M. Jørgensen, R. García-Valverde, S. Chaouki-Almagro, J. A. Villarejo, F. C. Krebs, Overcoming the scaling lag for polymer solar cells. *Joule* **1**, 274–289 (2017).

20. R. Søndergaard, M. Hösel, D. Angmo, T. T. Larsen-Olsen, F. C. Krebs, Roll-to-roll fabrication of polymer solar cells. *Mater. Today* **15**, 36–49 (2012).

21. J. Zhao, L.-W. Lo, Z. Yu, C. Wang, Handwriting of perovskite optoelectronic devices on diverse substrates. *Nat. Photon.* **17**, 964–971 (2023).

22. M. Saravanapavanantham, J. Mwaura, V. Bulović, Printed organic photovoltaic modules on transferable ultra-thin substrates as additive power sources. *Small Methods* **7**, e2200940 (2023).

23. D. Wang, L. Lu, Z. Zhao, K. Zhao, X. Zhao, C. Pu, Y. Li, P. Xu, X. Chen, Y. Guo, L. Suo, J. Liang, Y. Cui, Y. Liu, Large area polymer semiconductor sub-microwire arrays by coaxial focused electrohydrodynamic jet printing for high-performance OFETs. *Nat. Commun.* **13**, 6214 (2022).

24. K. Fukuda, Y. Takeda, Y. Yoshimura, R. Shiwaku, L. T. Tran, T. Sekine, M. Mizukami, D. Kumaki, S. Tokito, Fully-printed high-performance organic thin-film transistors and circuitry on one-micron-thick polymer films. *Nat. Commun.* **5**, 4147 (2014).

25. L. A. Ruiz-Preciado, S. Baek, N. Strobel, K. Xia, M. Seiberlich, S.-m. Park, U. Lemmer, S. Jung, G. Hernandez-Sosa, Monolithically printed all-organic flexible photosensor active matrix. *Npj Flex. Electron.* **7**, 6 (2023).

26. R. Steim, F. R. Kogler, C. J. Brabec, Interface materials for organic solar cells. *J. Mater. Chem.* **20**, 2499–2512 (2010).

27. B. R. Lee, E. D. Jung, J. S. Park, Y. S. Nam, S. H. Min, B.-S. Kim, K.-M. Lee, J.-R. Jeong, R. H. Friend, J.-S. Kim, S. O. Kim, M. H. Song, Highly efficient inverted polymer light-emitting diodes using surface modifications of ZnO layer. *Nat. Commun.* **5**, 4840 (2014).

28. O. Ostroverkhova, Organic optoelectronic materials: Mechanisms and applications. *Chem. Rev.* **116**, 13279–13412 (2016).

29. A. K. Bansal, S. Hou, O. Kulyk, E. M. Bowman, I. D. W. Samuel, Wearable organic optoelectronic sensors for medicine. *Adv. Mater.* **27**, 7638–7644 (2015).

30. H. Lee, E. Kim, Y. Lee, H. Kim, J. Lee, M. Kim, H.-J. Yoo, S. Yoo, Toward all-day wearable health monitoring: An ultralow-power, reflective organic pulse oximetry sensing patch. *Sci. Adv.* **4**, eaas9530 (2018).

31. C. M. Lochner, Y. Khan, A. Pierre, A. C. Arias, All-organic optoelectronic sensor for pulse oximetry. *Nat. Commun.* **5**, 5745 (2014).

32. C. Fuentes-Hernandez, W.-F. Chou, T. M. Khan, L. Diniz, J. Lukens, F. A. Larrain, V. A. Rodriguez-Toro, B. Kippelen, Large-area low-noise flexible organic photodiodes for detecting faint visible light. *Science* **370**, 698–701 (2020).

33. H. Jinno, T. Yokota, M. Koizumi, W. Yukita, M. Saito, I. Osaka, K. Fukuda, T. Someya, Self-powered ultraflexible photonic skin for continuous bio-signal detection via air-operation-stable polymer light-emitting diodes. *Nat. Commun.* **12**, 2234 (2021).

34. Y. Khan, D. Han, A. Pierre, J. Ting, X. Wang, C. M. Lochner, G. Bovo, N. Yaacobi-Gross, C. Newsome, R. Wilson, A. C. Arias, A flexible organic reflectance oximeter array. *Proc. Natl. Acad. Sci. U.S.A.* **115**, E11015–E11024 (2018).

35. D. Han, Y. Khan, J. Ting, S. M. King, N. Yaacobi-Gross, M. J. Humphries, C. J. Newsome, A. C. Arias, Flexible blade-coated multicolor polymer light-emitting diodes for optoelectronic sensors. *Adv. Mater.* **29**, 1606206 (2017).

36. I. Deckman, P. B. Lechêne, A. Pierre, A. C. Arias, All-printed full-color pixel organic photodiode array with a single active layer. *Org. Electron.* **56**, 139–145 (2018).

37. A. Wadsworth, Z. Hamid, J. Kosco, N. Gasparini, I. McCulloch, The bulk heterojunction in organic photovoltaic, photodetector, and photocatalytic applications. *Adv. Mater.* **32**, e2001763 (2020).

38. K. C. Tam, H. Saito, P. Maisch, K. Forberich, S. Feroze, Y. Hisaeda, C. J. Brabec, H.-J. Egelhaaf, Highly reflective and low resistive top electrode for organic solar cells and modules by low temperature silver nanoparticle ink. *Sol. RRL* **6**, 2100887 (2022).

39. W. Lee, H. Kim, I. Kang, H. Park, J. Jung, H. Lee, H. Park, J. S. Park, J. M. Yuk, S. Ryu, J.-W. Jeong, J. Kang, Universal assembly of liquid metal particles in polymers enables elastic printed circuit board. *Science* **378**, 637–641 (2022).

40. Q. Shen, M. Jiang, R. Wang, K. Song, M. H. Vong, W. Jung, F. Krisnadi, R. Kan, F. Zheng, B. Fu, P. Tao, C. Song, G. Weng, B. Peng, J. Wang, W. Shang, M. D. Dickey, T. Deng, Liquid metal-based soft, hermetic, and wireless-communicable seals for stretchable systems. *Science* **379**, 488–493 (2023).

41. H. Hu, H. Huang, M. Li, X. Gao, L. Yin, R. Qi, R. S. Wu, X. Chen, Y. Ma, K. Shi, C. Li, T. M. Maus, B. Huang, C. Lu, M. Lin, S. Zhou, Z. Lou, Y. Gu, Y. Chen, Y. Lei, X. Wang, R. Wang, W. Yue, X. Yang, Y. Bian, J. Mu, G. Park, S. Xiang, S. Cai, P. W. Corey, J. Wang, S. Xu, A wearable cardiac ultrasound imager. *Nature* **613**, 667–675 (2023).

42. D. J. Lipomi, B. C.-K. Tee, M. Vosgueritchian, Z. Bao, Stretchable organic solar cells. *Adv. Mater.* **23**, 1771–1775 (2011).

43. Y. Qian, X. Zhang, L. Xie, D. Qi, B. K. Chandran, X. Chen, W. Huang, Stretchable organic semiconductor devices. *Adv. Mater.* **28**, 9243–9265 (2016).

44. J. Wang, K. Fukuda, D. Inoue, D. Hashizume, L. Sun, S. Xiong, T. Yokota, T. Someya, Solution-processed electron-transport layer-free organic photovoltaics with liquid metal cathodes. *ACS Appl. Mater. Interfaces* **14**, 14165–14173 (2022).

45. T. Daeneke, K. Khoshmanesh, N. Mahmood, I. A. de Castro, D. Esrafilzadeh, S. J. Barrow, M. D. Dickey, K. Kalantar-zadeh, Liquid metals: Fundamentals and applications in chemistry. *Chem. Soc. Rev.* **47**, 4073–4111 (2018).

46. R. A. Bilodeau, D. Y. Zemlyanov, R. K. Kramer, Liquid metal switches for environmentally responsive electronics. *Adv. Mater. Interfaces* **4**, 1600913 (2017).

47. J.-H. Kim, S. Kim, H. Kim, S. Wooh, J. Cho, M. D. Dickey, J.-H. So, H.-J. Koo, Imbibition-induced selective wetting of liquid metal. *Nat. Commun.* **13**, 4763 (2022).

48. L. Cademartiri, M. M. Thuo, C. A. Nijhuis, W. F. Reus, S. Tricard, J. R. Barber, R. N. S. Sodhi, P. Brodersen, C. Kim, R. C. Chiechi, G. M. Whitesides, Electrical resistance of Ag^{TS}–$S(CH_2)_{n-1}CH_3$//Ga_2O_3/EGaIn tunneling junctions. *J. Phys. Chem. C* **116**, 10848–10860 (2012).

49. T. V. Neumann, M. D. Dickey, Liquid metal direct write and 3D printing: A review. *Adv. Mater. Technol.* **5**, 2000070 (2020).

50. K. Doudrick, S. Liu, E. M. Mutunga, K. L. Klein, V. Damle, K. K. Varanasi, K. Rykaczewski, Different shades of oxide: From nanoscale wetting mechanisms to contact printing of gallium-based liquid metals. *Langmuir* **30**, 6867–6877 (2014).

51. R. Su, S. H. Park, X. Ouyang, S. I. Ahn, M. C. McAlpine, 3D-printed flexible organic light-emitting diode displays. *Sci. Adv.* **8**, eabl8798 (2022).

52. Q. Xu, E. Brown, H. M. Jaeger, Impact dynamics of oxidized liquid metal drops. *Phys. Rev. E Stat. Nonlin. Soft Matter Phys.* **87**, 043012 (2013).

53. W. Kong, N. U. H. Shah, T. V. Neumann, M. H. Vong, P. Kotagama, M. D. Dickey, R. Y. Wang, K. Rykaczewski, Oxide-mediated mechanisms of gallium foam generation and stabilization during shear mixing in air. *Soft Matter* **16**, 5801–5805 (2020).

54. Y. Jiang, T. Liu, Y. Zhou, Recent advances of synthesis, properties, film fabrication methods, modifications of poly(3,4-ethylenedioxythiophene), and applications in solution-processed photovoltaics. *Adv. Funct. Mater.* **30**, 2006213 (2020).

55. T.-H. Han, M.-R. Choi, S.-H. Woo, S.-Y. Min, C.-L. Lee, T.-W. Lee, Molecularly controlled interfacial layer strategy toward highly efficient simple-structured organic light-emitting diodes. *Adv. Mater.* **24**, 1487–1493 (2012).

56. Z. Lou, J. Tao, B. Wei, X. Jiang, S. Cheng, Z. Wang, C. Qin, R. Liang, H. Guo, L. Zhu, P. Müller-Buschbaum, H.-M. Cheng, X. Xu, Near-infrared organic photodetectors toward skin-integrated photoplethysmography-electrocardiography multimodal sensing system. *Adv. Sci.* **10**, e2304174 (2023).

57. S. Wei, A. Jiang, H. Sun, J. Zhu, S. Jia, X. Liu, Z. Xu, J. Zhang, Y. Shang, X. Fu, G. Li, P. Wang, Z. Xia, T. Jiang, A. Cao, X. Duan, Shape-changing electrode array for minimally invasive large-scale intracranial brain activity mapping. *Nat. Commun.* **15**, 715 (2024).

58. W. G. Chung, J. Jang, G. Cui, S. Lee, H. Jeong, H. Kang, H. Seo, S. Kim, E. Kim, J. Lee, S. G. Lee, S. H. Byeon, J.-U. Park, Liquid-metal-based three-dimensional microelectrode arrays integrated with implantable ultrathin retinal prosthesis for vision restoration. *Nat. Nanotechnol.* (2024).

59. A. Miyamoto, S. Lee, N. F. Cooray, S. Lee, M. Mori, N. Matsuhisa, H. Jin, L. Yoda, T. Yokota, A. Itoh, M. Sekino, H. Kawasaki, T. Ebihara, M. Amagai, T. Someya, Inflammation-free, gas-permeable, lightweight, stretchable on-skin electronics with nanomeshes. *Nat. Nanotechnol.* **12**, 907–913 (2017).

60. S. Cheng, Z. Lou, L. Zhang, H. Guo, Z. Wang, C. Guo, K. Fukuda, S. Ma, G. Wang, T. Someya, H.-M. Cheng, X. Xu, Ultrathin hydrogel films toward breathable skin-integrated electronics. *Adv. Mater.* **35**, e2206793 (2023).

61. A. Spanu, A. Mascia, G. Baldazzi, B. Fenech-Salerno, F. Torrisi, G. Viola, A. Bonfiglio, P. Cosseddu, D. Pani, Parylene C-based, breathable tattoo electrodes for high-quality bio-potential measurements. *Front. Bioeng. Biotechnol.* **10**, 820217 (2022).

62. M. R. Ram, K. V. Madhav, E. H. Krishna, N. R. Komalla, K. A. Reddy, A novel approach for motion artifact reduction in PPG signals based on AS-LMS adaptive filter. *IEEE Trans. Instrum. Meas.* **61**, 1445–1457 (2012).

63. D. Seok, S. Lee, M. Kim, J. Cho, C. Kim, Motion artifact removal techniques for wearable EEG and PPG sensor systems. *Front. Electron.* **2**, 685513 (2021).

64. G. H. Lee, H. Kang, J. W. Chung, Y. Lee, H. Yoo, S. Jeong, H. Cho, J.-Y. Kim, S.-G. Kang, J. Y. Jung, S. G. Hahm, J. Lee, I.-J. Jeong, M. Park, G. Park, I. H. Yun, J. Y. Kim, Y. Hong, Y. Yun, S.-H. Kim, B. K. Choi, Stretchable PPG sensor with light polarization for physical activity–permissible monitoring. *Sci. Adv.* **8**, eabm3622 (2022).

65. J. Noh, G.-U. Kim, S. Han, S. J. Oh, Y. Jeon, D. Jeong, S. W. Kim, T.-S. Kim, B. J. Kim, J.-Y. Lee, Intrinsically stretchable organic solar cells with efficiencies of over 11%. *ACS Energy Lett.* **6**, 2512–2518 (2021).

66. J.-W. Lee, G.-U. Kim, D. J. Kim, Y. Jeon, S. Li, T.-S. Kim, J.-Y. Lee, B. J. Kim, Intrinsically-stretchable, efficient organic solar cells achieved by high-molecular-weight, electro-active polymer acceptor additives. *Adv. Energy Mater.* **12**, 2200887 (2022).

67. F. Ongul, S. A. Yuksel, S. Bozar, G. Cakmak, H. Y. Guney, D. A. M. Egbe, S. Gunes, Vacuum-free processed bulk heterojunction solar cells with E-GaIn cathode as an alternative to Al electrode. *J. Phys. D Appl. Phys.* **48**, 175102 (2015).

68. V. T. H. Pham, T. K. Trinh, N. T. N. Truong, C. Park, Liquid eutectic GaIn as an alternative electrode for PTB7:PCBM organic solar cells. *Jpn. J. Appl. Phys.* **56**, 046501 (2017).

69. F. Ongul, Solution-processed inverted organic solar cell using V2O5 hole transport layer and vacuum free EGaIn anode. *Opt. Mater.* **50**, 244–249 (2015).

70. G. S. Ryu, J. You, V. Kostianovskii, E. B. Lee, Y. Kim, C. Park, Y. Y. Noh, Flexible and printed PPG sensors for estimation of drowsiness. *IEEE Trans. Electron. Dev.* **65**, 2997–3004 (2018).

71. I. Titov, M. Köpke, M. Gerken, Monolithic integrated OLED–OPD unit for point-of-need nitrite sensing. *Sensors* **22**, 910 (2022).

72. I. Titov, M. Köpke, N. C. Schneidewind, J. Buhl, Y. Murat, M. Gerken, OLED-OPD matrix for sensing on a single flexible substrate. *IEEE Sens. J.* **20**, 7540–7547 (2020).

73. Y. Lee, J. W. Chung, G. H. Lee, H. Kang, J.-Y. Kim, C. Bae, H. Yoo, S. Jeong, H. Cho, S.-G. Kang, J. Y. Jung, D.-W. Lee, S. Gam, S. G. Hahm, Y. Kuzumoto, S. J. Kim, Z. Bao, Y. Hong, Y. Yun, S. Kim, Standalone real-time health monitoring patch based on a stretchable organic optoelectronic system. *Sci. Adv.* **7**, eabg9180 (2021).
